# Electron spin resonance resolves intermediate triplet states in delayed fluorescence

**DOI:** 10.1038/s41467-021-24612-9

**Published:** 2021-07-26

**Authors:** Bluebell H. Drummond, Naoya Aizawa, Yadong Zhang, William K. Myers, Yao Xiong, Matthew W. Cooper, Stephen Barlow, Qinying Gu, Leah R. Weiss, Alexander J. Gillett, Dan Credgington, Yong-Jin Pu, Seth R. Marder, Emrys W. Evans

**Affiliations:** 1 Department of Physics, Cavendish Laboratory, J J Thomson Avenue, University of Cambridge, Cambridge, UK; 2 Centre for Advanced Electron Spin Resonance (CAESR), Department of Chemistry, University of Oxford, Inorganic Chemistry Laboratory, Oxford, UK; 3 RIKEN Center for Emergent Matter Science (CEMS), Saitama, Japan; 4 School of Chemistry and Biochemistry and Center for Organic Photonics and Electronics, Georgia Institute of Technology, Atlanta, GA USA; 5 Pritzker School of Molecular Engineering, University of Chicago, Chicago, IL USA; 6 Department of Chemistry, Swansea University, Swansea, UK

**Keywords:** Organic LEDs, Electronic properties and materials

## Abstract

Molecular organic fluorophores are currently used in organic light-emitting diodes, though non-emissive triplet excitons generated in devices incorporating conventional fluorophores limit the efficiency. This limit can be overcome in materials that have intramolecular charge-transfer excitonic states and associated small singlet-triplet energy separations; triplets can then be converted to emissive singlet excitons resulting in efficient delayed fluorescence. However, the mechanistic details of the spin interconversion have not yet been fully resolved. We report transient electron spin resonance studies that allow direct probing of the spin conversion in a series of delayed fluorescence fluorophores with varying energy gaps between local excitation and charge-transfer triplet states. The observation of distinct triplet signals, unusual in transient electron spin resonance, suggests that multiple triplet states mediate the photophysics for efficient light emission in delayed fluorescence emitters. We reveal that as the energy separation between local excitation and charge-transfer triplet states decreases, spin interconversion changes from a direct, singlet-triplet mechanism to an indirect mechanism involving intermediate states.

## Abstract (web summary)

Despite advances in the design of thermally activated delayed fluorescence (TADF) emitters for devices, the effect of spin interactions is not well understood. Here, the authors report the role of spin-vibronic coupling in TADF organic emitters using transient electron spin resonance spectroscopy.

## Introduction

Thermally activated delayed fluorescence (TADF) organic molecules convert electricity to light more efficiently than traditional organic fluorophores, making them attractive materials for organic light-emitting diodes (OLEDs)^1–6^. Spin statistics for OLEDs dictate that 75% of electrically-generated excitons populate triplet (spin = 1) states (T_1_), which, in the case of traditional fluorophores, limits electroluminescence (EL) efficiency since radiative de-excitation of these states to the singlet (spin = 0) ground state (S_0_) is spin-forbidden^7,8^. Current commercial OLED devices employ organometallic chromophores based on iridium and platinum as red and green emitters. In such materials, enhanced spin–orbit coupling from the heavy element promotes direct emission from triplet excitons (phosphorescence), allowing up to 100% internal quantum efficiency (IQE) for EL^9–11^. Blue emitters based on the same approach have remained elusive, particularly because the long lifetimes of high-energy triplet excitons lead to OLED degradation^12,13^. Consequently, blue OLEDs typically employ organic fluorophores, with an enhancement of EL IQE achieved using triplet-triplet annihilation (TTA, T_1_ + T_1_ → S_1_ + S_0_), which can harvest up to half of the triplet excitons for light emission via singlets^14–16^. TTA-based OLEDs can in principle achieve up to 62.5% EL IQE. Within this context, TADF emitters present an alternative approach to achieving 100% EL IQE in devices, thereby offering a step-change in performance for blue OLED technologies^17,18^.

In TADF devices, indirect emission from dark triplet states via bright singlet excitons (T_1_ → S_1_ → S_0_) is activated by ambient thermal energy. There is general agreement that TADF emitters should be designed to have small singlet-triplet exchange energies that promote rapid forward and reverse intersystem crossing (ISC) between singlet and triplet excitons; such small exchange energies can be engineered by spatial separation of the highest occupied and lowest unoccupied molecular orbitals (HOMO and LUMO)^1–6^. However, there is no agreement on the spin interactions and spin-flip mechanisms responsible for the singlet-triplet interconversion in fluorophores exhibiting TADF with intramolecular charge-transfer (CT) states^19–22^. Typical TADF emitters have low-lying singlet and triplet excitonic states with mixed local excitation (LE) and intramolecular CT character^21,23^. Emission from the predominantly CT singlet excitonic state (^1^CT) via prompt or delayed fluorescence is desirable. Therefore, the triplet excitons populating either CT or local excitation states (^3^CT and ^3^LE) must be efficiently converted to ^1^CT. ISC between the singlet and triplet manifolds of fluorophores can be driven by a spin-orbit coupling (SOC) mediated spin conversion, whereby an orbital transition between two states generates a torque capable of flipping an electron’s spin. Direct SOC (dependent only on the electronic character of the coupled states) will in general be suppressed between a singlet and triplet state if they possess similar orbital character (El Sayed’s Rule)^24,25^. Therefore, in order to achieve singlet-triplet ISC between two electronic states of the same orbital character (e.g. ^1^CT to ^3^CT) additional processes must occur, which are dependent on both the electronic character and nuclear coordinates of the coupled states. However, the presence of a significant change in orbital character upon spin-flip (e.g. ^1^CT to ^3^LE) is not in itself sufficient to guarantee rapid ISC and, furthermore, it has been shown that direct SOC alone cannot explain high rates of ISC observed^26–28^. Vibration-mediated SOC has been suggested to explain this discrepancy, and also to facilitate singlet-triplet conversion between states of similar orbital character^18,29^. Vibrational contributions from an additional, or ‘intermediate’, triplet state give rise to the ‘spin-vibronic mechanism’ for the efficient conversion of dark triplet excitons to emissive singlet excitons (reverse ISC) in TADF emitters^30–32^. Revealing the character and formation dynamics of intermediate triplet states is challenging, although critical to understanding the TADF mechanism^33,34^.

Here we use transient electron spin resonance (trESR) spectroscopy to study the role of spin-orbit coupling and vibrational perturbations in ISC for TADF. While optical spectroscopy has proved a powerful tool to examine the photophysics and spin-conversion rates in TADF^30,31,35,36^, trESR provides a complementary window into the underlying spin physics. TrESR is sensitive to paramagnetic states and can therefore probe the formation dynamics and coupling mechanisms of triplet excitons^37^, as highlighted by broad applications in studying triplet excitons in organic photovoltaics^38–40^, singlet fission^41–44^, photosensitizers^37,45–47^, and molecular electronics^48,49^. Because trESR is not sensitive to singlet excitons (due to their diamagnetism) it can only explicitly probe forward ISC. However, assuming microscopic reversibility, the triplet exciton formation dynamics revealed are applicable to TADF-relevant reverse ISC. We have previously shown using trESR that spin-orbit coupling interactions mediate ISC for two benchmark TADF molecules: 1,2,3,5-tetrakis(carbazol-9-yl)-4,6-dicyanobenzene (4CzIPN) and 1,2-bis(carbazol-9-yl)-4,5-dicyanobenzene (2CzPN)^20^. The trESR data suggested a dynamic picture of SOC-mediated ISC involving vibrational modes, although the excited-state ordering and coupling requirements for efficient ISC were not established. To reveal these requirements, here we have conducted trESR studies on a series of ten molecular organic fluorophores exhibiting delayed fluorescence with varying ^3^LE–^3^CT energy gaps; this allows us to probe the spin-vibronic mechanism directly and resolve the nature of intermediate triplet states. TrESR gives conclusive evidence that ^3^LE can be populated from the singlet manifold by the vibrational SOC ISC mechanism. However, when the ^3^LE–^3^CT gap is sufficiently small, trESR reveals that additional spin conversion pathways emerge, and ^3^CT is populated via the spin-vibronic ISC mechanism because of enhanced ^3^LE–^3^CT coupling. TrESR can crucially resolve these different ISC mechanisms, which are influenced by difficult to predict molecular properties beyond the excited-state character and energetics measured with optical spectroscopy. Our spin-sensitive measurements reveal direct evidence that distinct triplet excitonic states (T_1_ + T_2_) mediate the spin conversion responsible for delayed fluorescence. These findings uncover the fundamental mechanism of the spin-vibronic ISC pathway relevant in TADF emitters, providing detail on the nature of the process by probing distinct yet vibronically-accessible ^3^LE and ^3^CT states.

## Results

### Design and detection of multiple triplet excitonic states

**Fig. 1:**
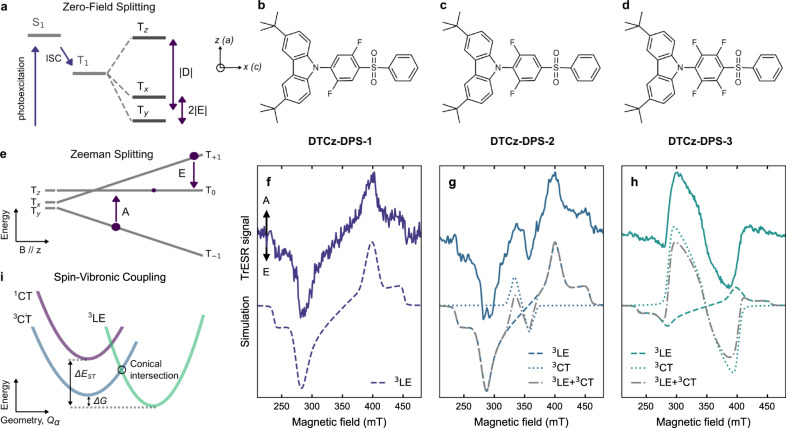
Molecular structures and electron spin resonance spectra. **a** Schematic diagram depicting the zero-field splitting (ZFS) of the lowest-lying triplet state (T_1_) into its three sublevels (T_*x*,*y*,*z*_). The magnitude of the ZFS parameters, *D* and *E*, describes the splitting of the triplet sublevels where *x*, *y*, and *z* are the ZFS axes along which spin density is distributed. The energetic spacing is not to scale. Molecular structures of (**b**) DTCz-DPS-1, (**c**) DTCz-DPS-2, and (**d**) DTCz-DPS-3. The in-plane molecular axes *a* and *c* and the ^3^LE ZFS axes *z* and *x* are shown. **e** Schematic diagram depicting the Zeeman energy separation of the triplet sublevels (low-field: T_*x*,*y*,*z*_; high-field: T_−1,0,+1_) when a magnetic field (B) is applied parallel to the *z* axis and the absorptive (A) and emissive (E) transitions that occur between sublevels when microwave radiation is resonant with energetic separation. Spin-polarised trESR signals collected at 30 K in toluene for (**f**) DTCz-DPS-1, (**g**) DTCz-DPS-2, and (**h**) DTCz-DPS-3. Solid lines show the trESR signal recorded 2 μs after 355 nm laser excitation and integrated over 1 μs. Dashed and dotted lines are the simulated local excitation triplet state (^3^LE) and charge-transfer triplet state (^3^CT) polarisation patterns, respectively. Dash-dot grey lines are the weighted sums of the ^3^LE and ^3^CT simulations. The spin-polarised patterns are characterised by absorptive (A) and emissive (E) features. **i** Spin-vibronic coupling of ^1^CT, ^3^LE and ^3^CT potential energy surfaces.

Three donor-acceptor emitters sharing a common 4-(3,6-di-*tert*-butyl-9*H*-carbazol-9-yl)diphenyl sulfone (DTCz-DPS) core structure were synthesised. We employed inductively electron-withdrawing fluorine substituents on a phenyl group linking carbazolyl and sulfone moieties to lower the LUMO energy of the DTCz-DPS core, resulting in lowered CT state energies as in our previous study^50^. The three emitters, DTCz-DPS-1, -2, and -3 (Fig. 1b–d) share a fixed ^3^LE energy level, associated with the carbazole donor, but possess increasingly red-shifted ^1^CT and ^3^CT, from DTCz-DPS-1 to -3, consistent with time-dependent density-functional theory (TD-DFT) calculations (Supplementary Table 1), described in ‘Methods’. The calculated Gibbs free energy difference between the ^3^LE and ^3^CT minima (the ^3^LE–^3^CT gap), in their optimised geometries, is reduced from DTCz-DPS-1 to -3 (Table 1). Importantly, our calculations also found that the direct SOC matrix elements between ^1^CT and ^3^LE are relatively constant for the three emitters (Table 1). Therefore, our structural modifications of excited-state energies allow higher-order SOC effects to be studied due to changing of the ^3^LE–^3^CT energy gap and energetic landscape, while direct SOC matrix elements between ^1^CT and ^3^LE are fixed.

**Table 1:** Experimental and TD-DFT calculated excited-state properties.

Compound	λ_PL_ (nm)^a^	S_1_ (eV)^b^	T_1_ (eV)^b^	τ_PF_ (ns)^c^	τ_DF_ (µs)^c^	% delayed^d^	H_SO_ (cm^−1^)^e^	ΔG (meV)^f^
DTCz-DPS-1	438	3.42	3.05	15	11	3	0.31	104
DTCz-DPS-2	449	3.40	3.05	17	31	28	0.27	81
DTCz-DPS-3	527	3.32	3.05	5	15	37	0.32	33

^a^Photoluminescence charge-transfer transition peak maxima measured in deoxygenated toluene at 295 K.

^b^The energetic onset of photoluminescence (S_1_) and phosphorescence (T_1_) in deoxygenated toluene at 77 K.

^c^Prompt fluorescence lifetime (±5 ns) and delayed fluorescence lifetime (±100 ns) from integrated photoluminescence kinetics, recorded in deoxygenated toluene at 295 K.

^d^Contribution of delayed fluorescence component to total photoluminescence from integrated time-resolved photoluminescence measured in deoxygenated toluene at 295 K.

^e^Root sum square of the *x*, *y*, *z* components of the calculated $|\langle {{\Psi }}_{1CT}|{\hat{H}}_{SO}| {{\Psi }}_{{3}L{{{{\rm{E}}}}}}\rangle$ at the optimised ^3^LE geometry.

^f^Difference in Gibbs free energy associated with ^3^LE and ^3^CT minima at their optimised geometries.

For the trESR measurements, pulsed laser excitation (355 nm) and cryogenic temperatures (30 K) were used to generate long-lived, molecular triplet excitons in dilute (500 μM), deoxygenated and flash-frozen toluene solutions. A variable applied magnetic field (200–500 mT) induced Zeeman energy separation of the triplet sublevels from the zero-field splitting (ZFS) eigenstates (T_*x*,*y*,*z*_, Fig. 1a, e). A continuous wave microwave source (~9.7 GHz) induced triplet sublevel transitions (absorptive, A, and emissive, E) when resonant with the sublevel energetic spacing; the intensity of these transitions is dependent on the triplet sublevel population (P_*x*,*y*,*z*_), which is determined by the nature of the triplet formation (ISC) process. The magnetic field positions of the positive (absorptive) and negative (emissive) peaks in the trESR signal (also known as ‘polarisation pattern turning points’) are determined by the ZFS parameters, *D* and *E* (Fig. 1a). The spin–spin dipolar interaction contribution to ZFS can yield a *D*-dependence on spin-density delocalisation across the molecule that allows for more LE- and CT-type triplets to be distinguished; a smaller *D*-value typically correlates with greater delocalisation, indicative of a CT state in the molecules investigated here^19,51^. *D*, *E*, and P_*x*,*y*,*z*_ are obtained from inspection, simulation and least-squares fitting (using EasySpin^52^) of the spin-polarised trESR signal. Following photoexcitation, the all-organic emitters have typically low triplet yield and hence data acquisition times of 4–12 h were required for each trESR spectrum.

Each emitter exhibited spin-polarised trESR signals with lifetimes of several microseconds, throughout which the spectral shapes were conserved. The trESR signal of DTCz-DPS-1 (Fig. 1f) has a 6-turning point EEEAAA polarisation pattern with a preferential population of T_*x*_ and T_*z*_ (P_*x*,*y*,*z*_ = 0.38, 0.00, 0.62). The ZFS parameters (|*D* | , | *E* | = 108, 9 mT), the position of the half-field transition (161 mT, Supplementary Fig. 4), and EEEAAA polarisation pattern of DTCz-DPS-1 are all typical of triplet states in organic chromophores^51^. These parameters are consistent with the ^3^LE associated with the carbazole monomer^53^, leading us to attribute the trESR signal to a triplet exciton localised on the carbazole donor. The trESR signals of DTCz-DPS-2 and -3 (Fig. 1g, h) show spin signatures of two overlapping triplet signals. It is evident from the line shape of the DTCz-DPS-2 trESR spectrum that the wider triplet signal resembles that seen for DTCz-DPS-1. Furthermore, the prominent half-field transition peak in the DTCz-DPS-2 trESR spectrum (162 mT, Supplementary Fig. 4) matches well with that in DTCz-DPS-1. After subtracting the ^3^LE contribution from the trESR signal of DTCz-DPS-2, the resulting triplet is narrower (|*D* | , | *E* | = 23, 3 mT) and has a spin-polarisation pattern that indicates relative overpopulation of T_*x*_ and T_*y*_ (P_*x*,*y*,*z*_ = 0.4, 0.6, 0.0). A reduced magnitude of *D* of this scale typically indicates an increased spatial separation of interacting spins; therefore, we attribute the narrow signal to the delocalised ^3^CT^19,51^.

The trESR signal of DTCz-DPS-3 (Fig. 1h) comprises a more intense central feature compared to DTCz-DPS-2. The total width of the DTCz-DPS-3 signal is unchanged and the same 161 mT half-field transition peak is recorded as in DTCz-DPS-1 (Supplementary Fig. 4); therefore, the same ^3^LE is assigned to the broad triplet signal. By subtracting the ^3^LE contribution from the total DTCz-DPS-3 trESR spectrum a narrower triplet signal ascribed to ^3^CT is obtained with P_*x*,*y*,*z*_ = 0.6, 0.4, 0.0, |*D* | , | *E* | = 56, 19 mT and a half-field transition peak at 169 mT; the breadth of the DTCz-DPS-3 ^3^CT signal compared to the DTCz-DPS-2 ^3^CT signal is a point to which we will later return. These results show that with decreasing ^3^LE–^3^CT gap (from DTCz-DPS-1 to -3), the recorded trESR spectra progress from a relatively pure ^3^LE signal to one resulting from contributions of both ^3^LE and ^3^CT polarisation patterns. The triplet ZFS simulation parameters of all three emitters are shown in Table 2.

**Table 2:** Zero-field splitting parameters (*D* and *E*) and spin sublevel populations (P_*x*,*y*,*z*_) of ^3^LE and ^3^CT obtained by simulation of the trESR signal detected at 30 K in deoxygenated toluene or by calculation of TD-DFT optimised excited-state geometries.

Compound	^3^LE Parameters				^3^CT Parameters			
	Experiment *D*, *E* (mT)	Calculated *D*, *E* (mT)	P_*x*,*y*,*z*_	Weight	Experiment *D*, *E* (mT)	Calculated *D*, *E* (mT)	P_*x*,*y*,*z*_	Weight
DTCz-DPS-1	−108, −9	−115, −10	0.38, 0.00, 0.62	1.0	/	/	/	/
DTCz-DPS-2	−110, −10	−110, −8	0.4, 0.0, 0.6	0.9	−23, −3	−76, −16	0.4, 0.6, 0.0	0.1
DTCz-DPS-3	−110, −8	−112, −9	0.4, 0.0, 0.6	0.2	−56, −19	−94, −17	0.6, 0.4, 0.0	0.8

The P_*x*,*y*,*z*_ uncertainty is ±0.1 in all cases except for DTCz-DPS-1 ^3^LE, which has an uncertainty ±0.01. The weighting factor corresponds to the contribution of each ^3^LE or ^3^CT simulation to its total simulated spectrum.

**Fig. 2:**
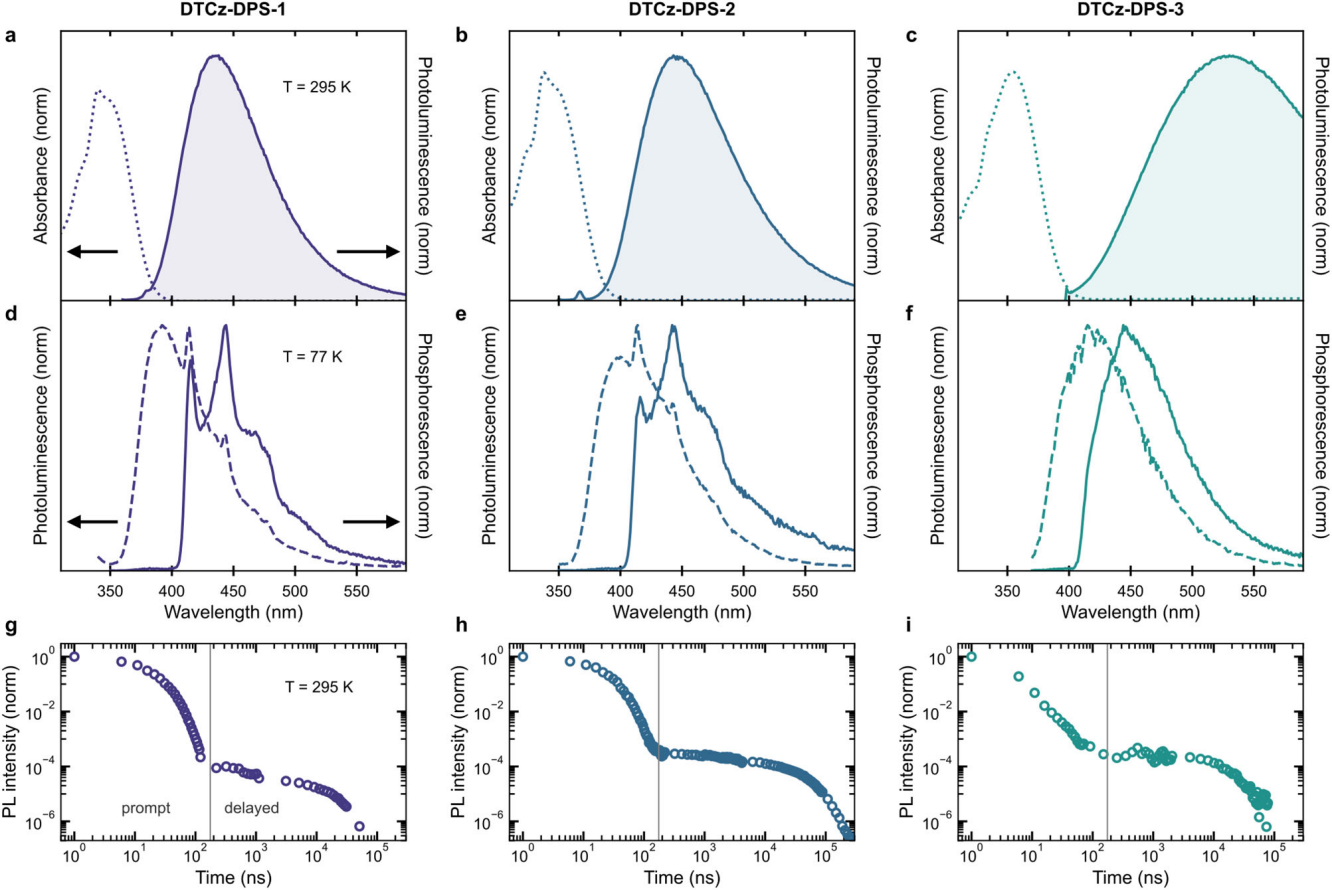
Photophysical spectra. Normalised absorbance (dotted line) and photoluminescence (filled line) spectra in deoxygenated toluene at 295 K (**a** DTCz-DPS-1, **b** DTCz-DPS-2, **c** DTCz-DPS-3). Normalised steady-state photoluminescence (dashed line) and gated (0.5 ms) phosphorescence (solid line) spectra in deoxygenated toluene at 77 K (**d** DTCz-DPS-1, **e** DTCz-DPS-2, **f** DTCz-DPS-3). Normalised integrated photoluminescence kinetics in deoxygenated toluene at 295 K (**g** DTCz-DPS-1, **h** DTCz-DPS-2, **i** DTCz-DPS-3).

Similarly, we observe the emergence of unstructured ^3^CT emission, in addition to structured ^3^LE emission, with decreasing ^3^LE–^3^CT gap in the frozen (77 K) toluene phosphorescence spectra (solid lines in Fig. 2d–f)^50,54^. We note that the phosphorescence was recorded at 77 K, which is a higher temperature than used in the trESR experiment (30 K). Crucially, both these temperatures are well below the freezing point of toluene (178 K). Therefore, the energetic relaxation of the ^1^CT states (a common phenomenon undergone by intramolecular CT states in solution) observed when comparing the photoluminescence (PL) in frozen (77 K) and solution (295 K) toluene (Fig. 2a–f) is minimised at both 30 K and 77 K. The progression in trESR and phosphorescence shows that ^3^LE and ^3^CT are both populated when the ^3^LE–^3^CT gap is sufficiently small^55^. Time-resolved PL measurements confirmed that all three of the emitters exhibit intramolecular CT character in prompt and delayed fluorescence at room temperature (295 K) in deoxygenated toluene (Fig. 2, Table 1, and Supplementary Fig. 10). The contribution of delayed fluorescence to the total emission was determined from the integrated time-resolved PL and is listed in Table 1. The electroluminescence performance of DTCz-DPS-1 and DTCz-DPS-3 were also evaluated in preliminary solution-processed OLEDs and are presented in the Supplementary Information (Supplementary Fig. 11 and Supplementary Table 7).

### Density-functional theory calculations

**Fig. 3:**
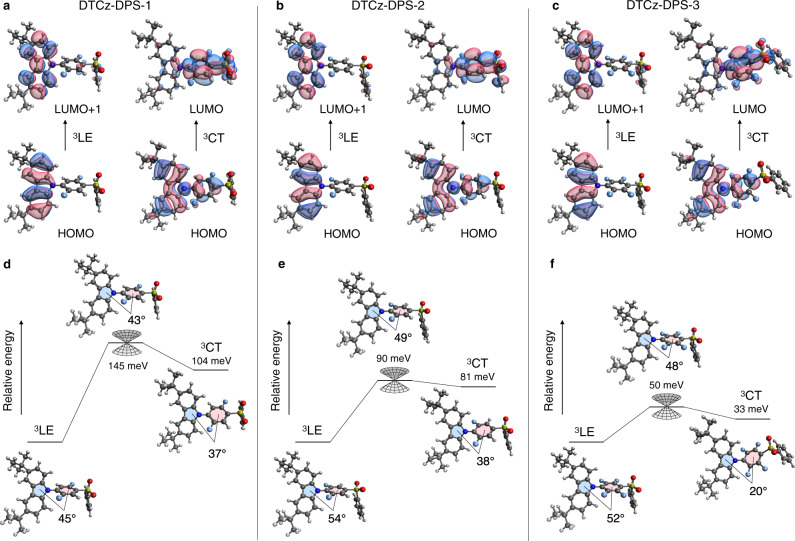
Molecular orbitals of relevant triplet excitonic states. The local excitation triplet state (^3^LE) and charge-transfer triplet state (^3^CT) configurations and highest occupied and lowest unoccupied molecular orbitals (HOMO and LUMO) of (**a**) DTCz-DPS-1, (**b**) DTCz-DPS-2, and (**c**) DTCz-DPS-3. The molecular geometry, relative Gibbs free energy, and donor–acceptor dihedral angle of (**d**) DTCz-DPS-1, (**e**) DTCz-DPS-2, and (**f**) DTCz-DPS-3 in the ^3^LE, ^3^CT configurations and at the minimum-energy conical intersection where two electronic triplet states are equal in energy and electronically coupled.

Optimised geometries and molecular orbitals were established for the low-lying excitonic states of DTCz-DPS-1, -2, and -3 (Fig. 3a–c) using the range-separated LC-BLYP functional^56^. The calculations predicted both ^3^LE and ^3^CT in close proximity to the ^1^CT, supporting our experimental observation of both triplets being populated in trESR and PL measurements in frozen toluene. The relative energy and donor-acceptor dihedral angle was calculated in the ^3^LE, ^3^CT configurations, and also at the minimal-energy conical intersection where two electronic triplet states are equal in energy and electronically coupled (Fig. 3). The geometrical change between the ^3^LE and ^3^CT configuration is predominantly characterised by rotation around the donor-acceptor linker: see dihedral angle between shaded planes in Fig. 3d–f.

The ZFS tensors (*D* and *E* parameters) were calculated for each ^3^LE and ^3^CT (Table 2) using the DFT-based method reported by Neese et al.^57,58^ that considers contributions to ZFS from both spin–spin interactions and spin–orbit interactions (Supplementary Table 2). DFT predicted *D* < 0 in all cases, therefore, *D* was assumed to be negative in all trESR spectral simulations. The sign of *E* was chosen such that axes labelling conformed with molecular axes labelling convention, as opposed to ESR convention. The total *D* and *E* values calculated for the ^3^LE configurations are in good quantitative agreement with the values obtained from trESR measurements. The calculated contribution to total *D* from spin–spin interactions decreases from ^3^LE to ^3^CT, whereas the contribution from spin–orbit interactions does not decrease. The resulting ^3^CT total *D* and *E* values are overestimated in comparison to those obtained from trESR measurements, although the experimental observation that the ZFS *D*-value of DTCz-DPS-3 ^3^CT is larger than DTCz-DPS-2 ^3^CT is correctly predicted. The larger calculated *D*-value of DTCz-DPS-3 compared to DTCz-DPS-2 arises from a greater spin–spin interaction contribution to ZFS (Supplementary Table 2). Variation in spin–spin interaction can be rationalised by examining the ^3^CT molecular orbitals in Fig. 3: the electron-withdrawing effect of the additional fluorine atoms in DTCz-DPS-3 localises the LUMO more strongly on the fluorinated phenyl ring than in DTCz-DPS-2 where the LUMO is more delocalised over both phenyl rings. Closer average HOMO-LUMO proximity leads to reduced spin-spin distance and enhanced interaction, which typically manifests as larger *D*-value.

### Further transient electron spin resonance studies: molecular modifications and concentration series

**Fig. 4:**
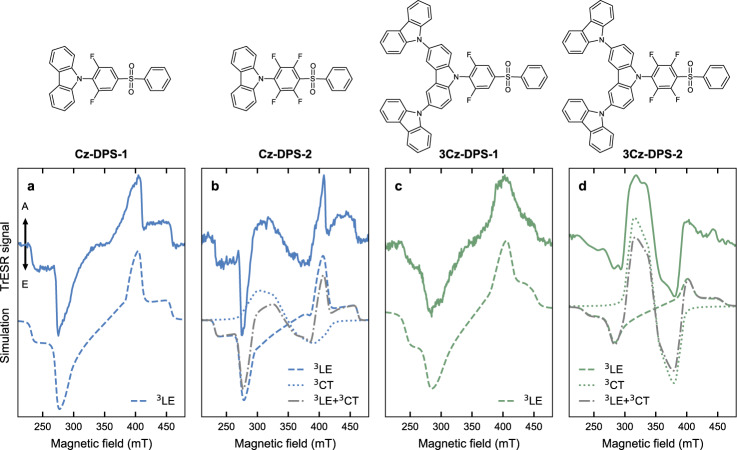
Electron spin resonance of further modified emitters. Molecular structures and spin-polarised trESR signal collected at 30 K in deoxygenated toluene of (**a**) Cz-DPS-1, (**b**) Cz-DPS-2, (**c**) 3Cz-DPS-1, and (**d**) 3Cz-DPS-2. Solid lines show the trESR signal, recorded 2 μs after 355 nm laser excitation and integrated over 1 μs. Dashed and dotted lines are the simulated local excitation triplet state (^3^LE) and charge-transfer triplet state (^3^CT) polarisation patterns, respectively. Dash-dot grey lines are the weighted sums of the ^3^LE and ^3^CT simulations.

We designed a number of additional experiments to test our interpretation that the trESR signals comprise polarisation patterns arising from ^3^LE and ^3^CT excitonic molecular states. First, we explored the effect of molecular substitutions on excited-state energies and subsequently the photophysics and ISC mechanism. We modified the donor groups of DTCz-DPS-2 and DTCz-DPS-3 by removing the tertiary butyl groups (to give Cz-DPS-1 and -2, respectively), or by replacing the tertiary butyl groups with additional carbazole units (to give 3Cz-DPS-1 and -2, respectively). The ^3^LE–^3^CT gap was decreased by fluorination on going from Cz-DPS-1 to −2 and from 3Cz-DPS-1 to -2; we observed with decreasing gap: (a) emergence of the ^3^CT trESR signals (Fig. 4) and ^3^CT phosphorescence (Supplementary Fig. 7, 8) and (b) increase in contribution to total PL from delayed fluorescence. These observations are consistent with our findings for DTCz-DPS-1, -2, and -3. Photophysics and trESR simulation parameters can be found in the Supplementary Information (Supplementary Fig. 7, 8, and Supplementary Table 4, 5). Trends in the calculated excited-state energies and direct SOC matrix elements between ^1^CT and ^3^LE for Cz-DPS-1 and -2 and 3Cz-DPS-1 and -2 (Supplementary Table 6) are also consistent with the trends identified for DTCz-DPS-1, -2, and -3. In non- or partially-fluorinated versions of the emitters (Cz-DPS-0, DTCz-DPS-0, 3Cz-DPS-0) the CT states were blue-shifted, widening both the ^3^LE–^1^CT and ^3^LE–^3^CT gaps such that no substantial delayed component of the time-resolved PL was detected at room temperature (Supplementary Fig. 9). The trESR spectra of the fluorescent emitters (Cz-DPS-0, DTCz-DPS-0, and 3Cz-DPS-0) exhibit only EEEAAA polarisation patterns, with signal widths and half-field peaks consistent with the ^3^LE associated with monomer carbazole, as before (Supplementary Fig. 9). The effects of changing the molecular environment by switching the solvent from toluene to 2-methyltetrahydrofuran were compared for DTCz-DPS-1 and 3Cz-DPS-2 and are discussed in the Supplementary Information (Supplementary Fig. 6). Finally, to rule out intermolecular interactions we reduced the concentration of DTCz-DPS-1, DTCz-DPS-3, and 3Cz-DPS-2 in toluene from 500 μM to 20 μM. No significant differences in trESR signal were observed, supporting our interpretation of the mechanisms responsible for triplet state formation as all being intramolecular processes, rather than concentration-dependent aggregate states or intermolecular energy transfer (Supplementary Fig. 5).

## Discussion

TADF facilitated by spin-orbit coupling, as described in the ‘Introduction’, can be interpreted within the following framework of ISC mechanisms^26,59^:

(I) Direct spin–orbit coupling between ^1^CT and ^3^LE;
(II) Vibrational spin–orbit coupling between ^1^CT and ^3^LE;
(III) Spin-vibronic coupling between ^1^CT and ^3^CT via intermediate ^3^LE.

Vibrational modes can enhance ISC in (II) because spin–orbit coupling matrix elements for ^1^CT–^3^LE transitions depend on the nuclear degree of freedom, ${Q}_{\alpha }$. The vibronic coupling in (III) drives rapid internal conversion between ^3^CT and ^3^LE, equilibrating their population; ^3^LE is then coupled to ^1^CT via either (I) or (II)^26,30^. ^3^LE is considered an intermediate in the sequence of state formation: ^1^CT–^3^LE–^3^CT (Fig. 1i). Any initial population of ^1^LE is assumed to rapidly convert to ^1^CT, as will be discussed later. Here, the ^3^CT–^3^LE vibronic coupling in (III) is assigned to modes associated with the donor–acceptor linker^20,60,61^. These three ISC processes are expressed together by the sum of their first non-zero terms:

1 $${\hat{H}}_{{SO}}=\left\langle {\varPsi }_{^1{CT}}|{\hat{H}}_{{SO}}|{\varPsi }_{^3{LE}}\right\rangle$$

2 $$+\mathop{\sum}\limits_{\alpha }\frac{\partial \left\langle {\varPsi }_{^1{CT}}|{\hat{H}}_{{SO}}|{\varPsi }_{^3{LE}}\right\rangle }{\partial {Q}_{\alpha }}{Q}_{\alpha }$$

3 $$+\frac{\left\langle {\varPsi }_{^1{CT}}|{\hat{H}}_{{SO}}|{\varPsi }_{^3{LE}}\right\rangle \left\langle {\varPsi }_{^3{LE}}|{\hat{H}}_{{vib}}|{\varPsi }_{^3{CT}}\right\rangle }{{E}_{^1{CT}}-{E}_{^3{LE}}}$$

where $\varPsi$ is the molecular wavefunction of states coupled either by the spin–orbit (${\hat{H}}_{{SO}}$) or vibronic (${\hat{H}}_{{vib}}$) Hamiltonian^26^. The vibronic coupling between two states generally increases as the energy gap between the states decreases and hence mechanism (III) only significantly contributes to the full spin–orbit interaction if the ^3^LE–^3^CT gap (Δ*G*) is sufficiently small (<0.5 eV). Furthermore, orbital and vibrational mode-dependent mechanisms are determined by the geometry of a molecule and differently affect ISC mechanisms (I), (II), and (III)^22,25,26^. TrESR polarisation patterns are fingerprints for the relative spin population of the triplet ZFS sublevels (*x*, *y*, *z*), which are also related to the molecular geometry^62^—Fig. 1 shows how the ^3^LE ZFS axes are related to the molecular axes (*a, b, c*) in DTCz-DPS-1, -2, and -3. These relationships mean that triplet formation by each ISC mechanism will yield a particular trESR polarisation pattern that can be distinguished by the ISC mechanism’s orbital and/or vibrational mode dependence on molecular geometry.

TADF emitters generally comprise all-organic electron donor and acceptor units. Individually the donor and acceptor units, on which LE states are localised, typically possess at least a C_2_ symmetry axis; symmetry does not persist in the delocalised CT states. Under these symmetry conditions the triplet sublevel populations describing the trESR polarisation pattern for each ISC mechanism in a TADF emitter is as follows:

(I) Overpopulation along the C_2_ symmetry axis (*x* here);
(II) Overpopulation of the in-plane axes (*x* and *z* here);
(III) Redistribution of the population from the in-plane (*x* and *z* here) to the out-of-plane (*y* here) axes.

The difference between ISC mechanisms (II) and (III) is the CT-like transition from ^3^LE to ^3^CT upon internal conversion. In a donor–acceptor emitter, the electron density in the ^3^LE configuration is localised on either the acceptor or, in this case, the donor unit and hence the triplet ZFS (*x*, *y*, *z*) and molecular (*a*, *b*, *c*) axes will be well-aligned. In contrast, the electron density in a ^3^CT configuration will be delocalised across both the donor and acceptor units (Fig. 3). The donor-acceptor twist, integral to TADF design, results in the rotation of the ^3^CT ZFS axes relative to the ^3^LE ZFS axes^63^. Therefore, the spin-density redistribution upon charge-transfer internal conversion from ^3^LE to ^3^CT rotates the triplet ZFS axes such that overpopulation of the out-of-plane axes (relative to the ^3^LE ZFS axes) can arise. This additional out-of-plane population causes the difference between the population of T_*x*,*y*,*z*_ in ISC mechanism (II) and (III)^64,65^. The donor–acceptor twist is a fundamental TADF molecular design rule because the spatial separation of HOMO and LUMO is required to reduce singlet-triplet offset and enable reverse ISC. Therefore, we can expect the relationship between molecular structure, ISC mechanism, and trESR pattern to be applicable to many classes of TADF emitters, as well as to donor–acceptor CT-type molecules in general.

Applying the framework outlined above to the triplet sublevel populations from experimental spectra (Table 2), we can infer the ISC mechanisms responsible for the population of ^3^LE and ^3^CT in each emitter. The ^3^LE states of DTCz-DPS-1, -2, and -3 (and Cz-DPS-1 and -2, 3Cz-DPS-1 and -2) are populated via vibrational SOC with the photoexcited ^1^CT – mechanism (II) – apparent from the overpopulation of T_*x*_ and T_*z*_^45,51,62,66,67^. On the other hand, the triplet sublevel corresponding to the out-of-plane molecular axis (T_*y*_) is relatively overpopulated in the ^3^CT polarisation patterns of DTCz-DPS-2 and -3 (and Cz-DPS-2 and 3Cz-DPS-2). The distinct triplet trESR spectra, therefore, reveal that spin-vibronic coupling between ^1^CT and ^3^CT via intermediate ^3^LE – mechanism (III) – is responsible for ^3^CT population^20,59,60^. The ability to resolve these ISC mechanisms from one another is a crucial advantage of using trESR; it is not possible to predict how excited states interact based purely on optical measurements of their separation energies or excitation character because additional factors arising from molecular structure affect ISC.

Our computational results qualitatively support the experimental triplet sublevel population assignments of the trESR spectra, and the involvement of vibrational and vibronic enhancement to SOC. First, the calculated $\left\langle {\varPsi }_{^1{CT}}|{\hat{H}}_{{SO}}|{\varPsi }_{^3{LE}}\right\rangle$ at the optimised ^3^LE geometries (described in the ‘Methods’) confirms that if direct SOC between ^3^LE and ^1^CT – mechanism (I) – were the dominant mechanism we would indeed observe overpopulation predominantly in the ^3^LE *x* plane (Supplementary Table 3, 6). The DFT calculations also predict the relationship between the ^3^LE ZFS and in-plane molecular axes (overpopulation in T_*x*_ and T_*z*_). A rotation of the in-plane *x*–*z* frame upon charge-transfer internal conversion would redistribute population from T_*z*_ to T_*y*_, consistent with the experimental observations (Table 2). The most significant mode identified in the calculations that are associated with the ^3^LE–^3^CT conversion is the rotation around the single bond between the DTCz donor and DPS acceptor in DTCz-DPS-1, -2, and -3 (Fig. 3d–f); this torsional mode likely drives vibronic coupling^20^.

The *D* and *E* values predicted by DFT for ^3^LE in each emitter agree well quantitively with those obtained from the measured trESR (Table 2). However, only the qualitative trend in relative magnitude is well-predicted in DFT calculations of ^3^CT; the calculated *D* and *E* values are overestimated compared to those obtained from the measured trESR (Table 2). As discussed, we would expect spin–orbit interactions related to ^3^CT configurations to be weaker compared to their ^3^LE counterparts; no such reduction in the contribution to *D* from spin–orbit interactions is predicted in the DFT calculations (Supplementary Table 2). We suggest that the mismatch between predicted and measured ^3^CT *D* and *E* values is a result of this overestimation of spin–orbit interactions between ^3^CT and other nearby electronic states. This discrepancy highlights the current limitations of common computational calculations and the necessity for experimental trESR to reveal the complex nature of multiple triplet states, which underpin the spin physics of TADF-type organic emitters.

To reiterate: our findings show that it is indirect, not direct, coupling of ^3^CT to the singlet manifold that facilitates ISC. This assertion is supported by the following pieces of evidence, which rule out direct ISC between ^3^CT and either ^1^CT or ^1^LE. Direct ISC from ^1^CT to ^3^CT would need to be mediated by hyperfine coupling because the lack of orbital change suppresses SOC. Hyperfine-mediated ISC facilitates the transfer from the singlet state to only the energetically closest high-field triplet sublevel and consequently has a trESR polarisation pattern that is distinct from the SOC-mediated ISC patterns described above, and it is not observed in our measurements^19,39^. If ^1^LE to ^3^CT ISC were prevalent, we would not observe a dependence of the ^3^CT trESR signal on the ^3^LE–^3^CT gap, and would instead observe the ^3^CT spectra in all measurements, which we do not. Further, we would not expect a substantial population of ^1^LE for two reasons. First, ^1^LE to ^1^CT internal conversion is rapid (ps) compared with ISC (ns)^22,45,68–71^. Second, in the experiment, the ^1^CT absorption band was selectively excited (and is spectrally distinct from the ^1^LE absorption) in order to mimic operational OLED conditions where the lowest energy excitonic states are electrically generated (Fig. 2a–c solid lines and Supplementary Table 1). To summarise, the progression from a purely ^3^LE polarisation pattern to a combination of both ^3^LE and ^3^CT polarisation patterns with decreasing ^3^LE–^3^CT gap is evidence that a small ^3^LE–^3^CT gap regime exists in which strong vibronic coupling persists, even at low temperatures (kT < 3 meV), such that ^3^LE and ^3^CT are simultaneously populated. It is worth noting that, as TADF development is predominantly focused on the critical task of achieving efficient blue emission, the above outlined spin-vibronic mechanism is described in the context of the energetic regime attainable in blue emitters and shown in Fig. 1i: ^1^CT > ^3^CT > ^3^LE^6,22^. However, the same principles can be applied to a donor–acceptor emitter exhibiting a different excited-state ordering. We note that the unusual observation of multiple, distinct triplet signals in trESR spectra has also been reported for twisted donor–acceptor triplet photosensitizers, although the mechanism through which both triplets were populated was not rationalised;^45–47^ we postulate that the spin-vibronic mechanism may be active in such triplet photosensitizers.

Finally, we return to discuss the photophysical results in light of the spin-vibronic mechanism determined from trESR. The increase of ^3^CT spectral contribution with decreasing ^3^LE–^3^CT gap observed here (and in other systems^55,69,72,73^) reinforces the direct evidence for the spin-vibronic mechanism from trESR. Furthermore, the emergence of the additional ^3^CT trESR spectra, reflecting the strength of vibronic coupling, correlates with increased contribution to total emission from delayed fluorescence. However, as the ^3^LE–^3^CT gap decreases so does the ^3^LE–^1^CT gap and therefore their combined effect on delayed fluorescence cannot be decoupled. We note that the dynamics of excited state couplings are also affected by time- and temperature-dependent molecular reorganisation, as is evident when comparing the 295 K fluorescence with the 77 K fluorescence (dashed lines in Fig. 2a–c versus dashed lines in Fig. 2d–f). The spectral redshift undergone by charge-transfer states from 77 K to 295 K (due to post-photoexcitation molecular reorganisation) increases from DTCz-DPS-1 to -3. The differing reorganisation complicates the temporal evolution of energetic spacing between ^1^CT, ^3^LE, and ^3^CT, and hence coupling dynamics at room temperature are not directly comparable to frozen solution measurements.

TrESR has been used previously to probe triplet excitons in twisted donor-acceptor emitters that, due to their large T_1_–T_2_ energy gap (>0.6 eV), are non-interacting and exhibit delayed fluorescence through either the hot-exciton^74^ or hyperfine pathways^75^. Our experimental results for the present series of donor–acceptor molecules, supported by computational calculations, show that coupling between the singlet and triplet manifold is facilitated by the spin-vibronic mechanism when ^3^LE is in energetic proximity (<100 meV) to ^3^CT. This coupling drives ISC, and therefore TADF-relevant reverse ISC (assuming microscopic reversibility). We have demonstrated that trESR can reveal mechanistic details of ISC, providing a critical, complementary approach to optical spectroscopy methods^33,76,77^ and have established a trESR framework to study the spin-vibronic mechanism in TADF emitters. We have demonstrated that the mechanism for ISC within an emitter can be tuned through chemical modifications; engineering and measuring the spin properties are critical for the informed development of efficient TADF emitters. We have characterised the nature of triplet excitations in the chemically modified set of delayed fluorescence emitters, resolving two distinct triplet excitonic states (T_1_ and T_2_) involved in spin-vibronic coupling. This distinction supports the basis for achieving spin-vibronic TADF by engineering distinct yet vibronically-accessible ^3^LE and ^3^CT states. As discussed, the mechanism of ^3^CT population by vibronic coupling with ^3^LE may find further application in the development of heavy-atom free triplet photosensitizer design^45–47^. Tuning spin–orbit coupling in molecular systems and their subsequent trESR signatures, as demonstrated here, is not only of paramount importance to blue OLED development but may also find resonance more broadly in the development of organic materials for light-controlled quantum information and spintronics^78–82^.

## Methods

### Synthesis of emitters

The emitters were generally synthesised by nuclophilic substitution reactions between the appropriate carbazole derivatives and partially fluorinated derivatives of diphenylsulfone in dimethylsulfoxide in the presence of potassium carbonate, as described in the detail in the Supplementary Information. The fluorinated diphenylsulfones were obtained by reaction of lithio(fluoroarenes), obtained through bromine-lithium exchange or deprotonation, with 1,2-diphenyldisulfane, followed by oxidation with 3-chlorobenzoperoxoic acid.

### Transient electron spin resonance spectroscopy

Dilute solutions were prepared at 500 μM in toluene in a nitrogen environment. The solutions were contained within 3.8 mm O.D. clear-fused quartz tubes and degassed by 4 freeze-pump-thaw cycles before being flame-sealed. Measurement of transient continuous wave ESR (trESR) was done in the Centre for Advanced ESR (CAESR) in the Department of Chemistry of the University of Oxford, using a Bruker BioSpin EleXSys I E680 at X-band (9.7 GHz, 0.2 mW) with an ER 4118X-MD5W resonator. The temperature was controlled with an Oxford Instruments CF935O cryostat under liquid helium flow and an ITC-503S controller. An Opotek Opolete HE355 LASER was used for optical excitation of the samples and it was synchronised to the spectrometer by a Stanford Research DG645 delay generator. Use of a Stanford Research SR560 low-noise preamplifier of the Schottky diode-detected CW Mode signal at 3–300 kHz was in substitute of the video amplifier, following verification against the quadrature mix-down DC-AFC Transient Mode signal of 20 MHz bandwidth setting. Spectra were recorded for between 4 and 12 h. Data was processed with MatLab (The Mathworks, Natick, N.J.) and ESR simulations made use of the EasySpin routines^53^.

### Density-functional theory calculations

The linear-response TD-DFT calculations were performed using the LC-BLYP/6-31 G(d) within the Tamm–Dancoff approximation^83^ implemented in the Gaussian 16 programme. The geometry optimisation of the minimum-energy conical intersection between the ^3^LE and the ^3^CT was performed with the GRRM17 programme^84^, which refers to the energy and gradient calculated by the Gaussian 16 programme. $\left\langle {\varPsi }_{^1{CT}}|{\hat{H}}_{{SO}}|{\varPsi }_{^3{LE}}\right\rangle$ was calculated using the TD-DFT LC-BLYP/6-31 G(d) with the scalar relativistic zeroth-order regular approximation (ZORA) Hamiltonian^85,86^ implemented in the ORCA 4.2.1 programme. The ZFS parameters of the ^3^LE and the ^3^CT were simulated using the DFT PBE0/6–31 G(d) with the spin-spin term calculated from the spin-densities derived from the unrestricted natural orbitals^57^ and the spin-orbit term calculated by the coupled-perturbed SOC approach^58^ implemented in the ORCA 4.2.1 programme.

### Steady-state absorption and emission spectra

Dilute solutions were prepared at 100 μM in toluene. UV−vis absorption spectra were measured using a Cary 5000UV−vis−NIR spectrophotometer. PL spectra were recorded on a Horiba FL3-2i Fluorometer equipped with a liquid nitrogen attachment in solution at ambient temperature or either as steady-state or gated spectra at 77 K.

### Time-resolved photoluminescence

Dilute solutions were prepared at 100 μM in deoxygenated toluene in a nitrogen environment and subsequently measured in a nitrogen environment. Time-resolved PL spectra were recorded using an electrically-gated intensified charge-coupled device (ICCD) camera (Andor iStar DH740 CCI-010) connected to a calibrated grating spectrograph (Andor SR303i). Pulsed 325 nm photoexcitation was provided at a repetition rate of 1 kHz. Temporal evolution of the PL was obtained by stepping the ICCD gate delay with respect to the excitation pulse. The minimum gate width of the ICCD was 5 ns. Recorded data was subsequently corrected to account for camera sensitivity.

## Supplementary information

Supplementary Information

## Data Availability

The data underlying this article are available at: 10.6084/m9.figshare.14428766.

## Code availability

Codes used to analyse data in this manuscript are available from the corresponding author upon reasonable request.
