# Supplementary material for: Electron spin resonance resolves intermediate triplet states in delayed fluorescence
Source: Nat Commun. 2021 Jul 26;12:4532. doi: 10.1038/s41467-021-24612-9 (PMC8313702; doi:10.1038/s41467-021-24612-9)
Supplement: Supplementary file 1 — Supplementary Information [file 41467_2021_24612_MOESM1_ESM.pdf]

Supplementary Information for Electron spin resonance resolves intermediate triplet states in delayed fluorescence

Contents

Synthesis 2
Supplementary Figure 1. 3
Supplementary Figure 2. 4
Density functional theory calculations 13
Supplementary Table 1. 13
Supplementary Table 2. 13
Supplementary Table 3. 13
Electron spin resonance 14
Supplementary Figure 4. 14
Supplementary Figure 5. 15
Supplementary Table 4. 15
Supplementary Figure 6. 16
Supplementary Figure 7. 17
Additional compounds: trESR, photophysics and DFT 17
Supplementary Figure 8. 18
Supplementary Figure 9. 19
Supplementary Table 5. 20
Supplementary Table 6. 20
Photophysics 21
Supplementary Figure 10. 21
Electroluminescence performance 21
Supplementary Table 7. 21
Supplementary Figure 11. 22

Synthesis

All reagents and anhydrous solvents were purchased from commercial sources and were used without further purification unless otherwise stated. The detailed synthetic procedures and characterization data for the intermediates and the final products are shown in Figure S1-S3. ^{1}H, ^{13}C, and ^{19}F NMR spectra were recorded in $CDCl_3$ using a Bruker AVANCE III HD 500 (operating at 500, 125, and 470.4 MHz, respectively), a Bruker AVANCE III 400, or a Varian Mercury Vx400 (operating at 400, 100, and 376.5 MHz, respectively). The signals were referenced to the solvent peak (7.27 ppm for the ^{1}H NMR signal of residual $CHCl_3$ and 77.0 ppm for the ^{13}C NMR signal of $CDCl_3$), to tetramethylsilane as an internal standard (0.00 ppm for both ^{1}H and ^{13}C NMR signals), or, for ^{19}F NMR, using a capillary insert containing trifluoroacetic acid as an external standard (−76.55 ppm). Column chromatography was carried out using silica gel (60 Å, 40−63μm) as the stationary phase. Mass spectra were measured on an Applied Biosystems 4700 Proteomics Analyzer using MALDI or a VG Instruments 70-SE using electron impact (EI) mode. Elemental analyses were performed by Atlantic Microlabs.

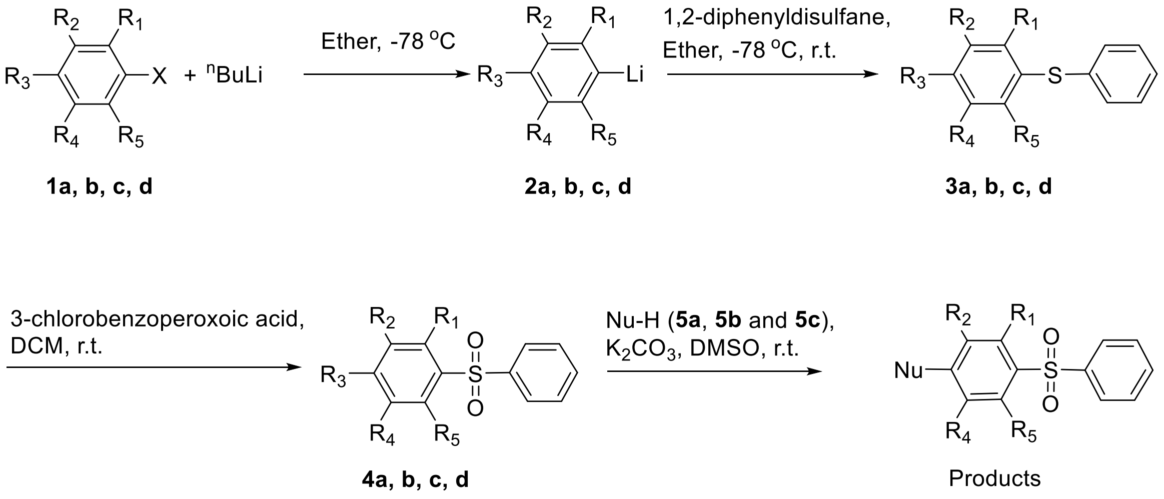

1a: R_1 = -F, R_2 = -H, R_3 = -F, R_4 = -F, R_5 = -H, X = -Br
1b: R_1 = -H, R_2 = -F, R_3 = -F, R_4 = -F, R_5 = -H, X = -Br
1c: R_1 = -F, R_2 = -H, R_3 = -F, R_4 = -H, R_5 = -F, X = -Br
1d: R_1 = -F, R_2 = -F, R_3 = -F, R_4 = -F, R_5 = -F, X = -H

2a, 3a, 4a: R_1 = -F, R_2 = -H, R_3 = -F, R_4 = -F, R_5 = -H
2b, 3b, 4b: R_1 = -H, R_2 = -F, R_3 = -F, R_4 = -F, R_5 = -H
2c, 3c, 4c: R_1 = -F, R_2 = -H, R_3 = - F, R_4 = -H, R_5 = -F
2d, 3d, 4d: R_1 = -F, R_2 = -F, R_3 = -F, R_4 = -F, R_5 = -F

5a: Nu-H = 3,6-di-*tert*-butylcarbazole
5b: Nu-H = carbazole
5c: Nu-H = 3,6-di(carbazol-9-yl)carbazole

Products:
DTCz-DPS-0: R_1 = -F, R_2 = -H, Nu = 3,6-di-*tert*-butylcarbazol-9-yl, R_4 = -H, R_5 = -F
DTCz-DPS-1: R_1 = -F, R_2 = -H, Nu = 3,6-di-*tert*-butylcarbazol-9-yl, R_4 = -F, R_5 = -H
DTCz-DPS-2: R_1 = -H, R_2 = -F, Nu = 3,6-di-*tert*-butylcarbazol-9-yl, R_4 = -F, R_5 = -H
DTCz-DPS-3: R_1 = -F, R_2 = -F, Nu = 3,6-di-*tert*-butylcarbazol-9-yl, R_4 = -F, R_5 = -F
Cz-DPS-1: R_1 = -H, R_2 = -F, Nu = carbazol-9-yl, R_4 = -F, R_5 = -H
Cz-DPS-2: R_1 = -F, R_2 = -F, Nu = carbazol-9-yl, R_4 = -F, R_5 = -F
3Cz-DPS-1: R_1 = -H, R_2 = -F, Nu = 3,6-di(carbazol-9-yl)carbazol-9-yl, R_4 = -F, R_5 = -H
3Cz-DPS-2: R_1 = -F, R_2 = -F, Nu = 3,6-di(carbazol-9-yl)carbazol-9-yl, R_4 = -F, R_5 = -F

Supplementary Figure 1. **Synthesis of DTCz-DPS-0, DTCz-DPS-1, DTCz-DPS-2, DTCz-DPS-3, Cz-DPS-1, Cz-DPS-2, 3Cz-DPS-1, 3Cz-DPS-2.**

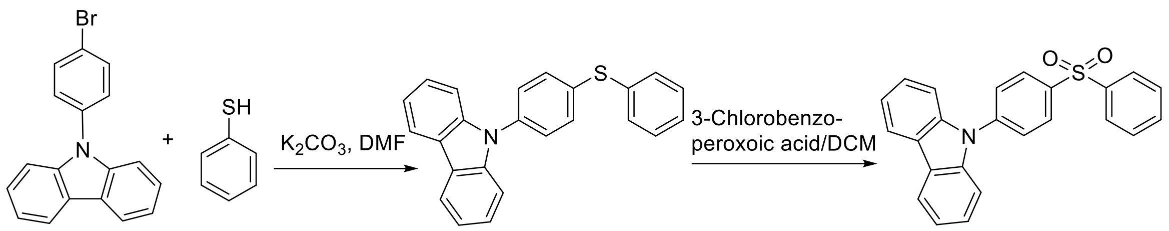

6 7 8 Cz-DPS-0

Supplementary Figure 2. **Synthesis of Cz-DPS-0.**

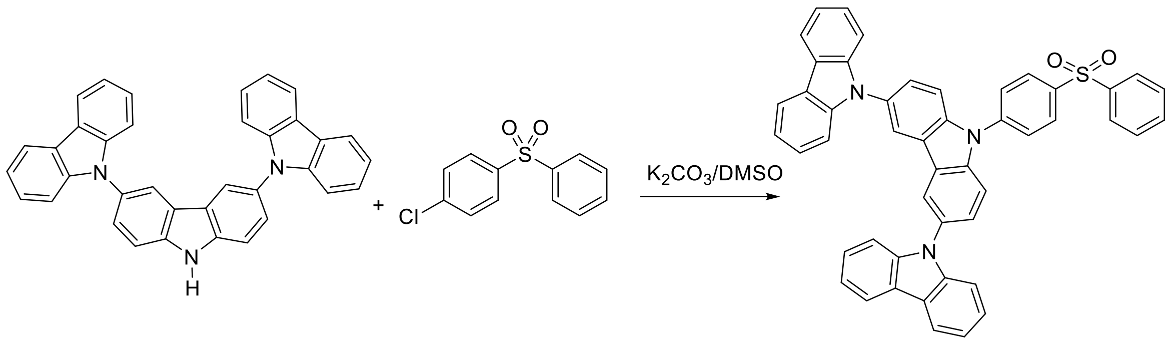

5c 9 3Cz-DPS-0

Supplementary Figure 3. **Synthesis of 3Cz-DPS-0.**

Phenyl(2,4,5-trifluorophenyl)sulfane (3a): ^{n}BuLi (18.0 mL of a 2.65 M in hexane, 48 mmol) was slowly added into a solution of 1-bromo-2,4,5-trifluorobenzene (10.0 g, 47.4 mmol) in dry ether (150 mL) at –78 °C under nitrogen. After the addition of ^{n}BuLi, the reaction mixture was stirred at –78 C for 1.5 h. 1,2-Diphenyldisulfane (10.0 g, 45.8 mmol) was added into the reaction mixture at –78 °C; the reaction mixture was then allowed to warm to room temperature and stirred for a further 2 h. Water (100 mL) was added, the product was extracted with pentane (100 mL), and washed with water (6 × 150 mL). The organic layer was dried over on Na_2SO_4, filtered, and evaporated to afford light yellow liquid 10.0 g (91% yield, assuming pure). This compound was used for the next step without further purification.

Phenyl(3,4,5-trifluorophenyl)sulfane (3b): 3b was synthesized using the same procedures described above for the synthesis of **3a,** but using 1-bromo-3,4,5-trifluorobenzene in place of 1-bromo-2,4,5-trifluorobenzene. The product was obtained as pale yellow solid in 86% yield. This compound was used for the next step without further purification.

Phenyl(2,4,6-trifluorophenyl)sulfane (3c): 3c was synthesized using the same procedures described above for the synthesis of **3a**, but using 1-bromo-2,4,6-trifluorobenzene in place of 1-bromo-2,4,5-trifluorobenzene. The product was obtained as pale yellow liquid in 96% yield. This compound was used for the next step without further purification.

(Perfluorophenyl)(phenyl)sulfane (3d): 3d was synthesized using the same procedures described above for the synthesis of **3a,** but using pentafluorobenzene in place of 1-bromo-2,4,5-trifluorobenzene. The product was obtained as a white solid in 92% yield. This compound was used for the next step without further purification.

1,2,4-Trifluoro-5-(phenylsulfonyl)benzene (4a): mCBPA (12.0 g, 77%, 69.5 mmol) was added to a stirred solution of phenyl(2,4,5-trifluorophenyl)sulfane (5.0 g, 21 mmol) in dichloromethane (60 mL) was added at room temperature under nitrogen. After stirring 24 h, the reaction mixture was purified by silica gel column using dichloromethane as eluent. After removal of the solvent, the product was recrystallized from acetone/water. White product solid was collected by filtration and washed with water. After drying under vacuum, the product was obtained as a white solid (5.02 g, 88%). ^{1}H NMR (400 MHz, $CDCl_3$) δ: 7.99 (m, 3H), 7.66 (m, 1H), 7.56 (m, 2H), 6.98 (m, 1H) ppm. ^{13}C{^{1}H} NMR (100 MHz, $CDCl_3$) δ: 154.94 (ddd, J_{1CF} = 255.8 Hz, J_{2CF} = 14.7 Hz, J_{3CF} = 4.5 Hz), 153.89 (dt, J_{1CF} = 259.5 Hz, J_{2CF} = 14.0 Hz), 146.55 (ddd, J_{1CF} = 250.0 Hz, J_{2CF} = 13.2 Hz, J_{3CF} = 3.7 Hz), 140.02, 134.14, 129.31, 128.16, 126.02

(d, J_{CF} = 16.1 Hz), 118.20 (d, J_{CF} = 22.0 Hz), 107.78 (dd, J_{1CF} = 27.1 Hz, J_{2CF} = 21.2 Hz) ppm. ^{19}F NMR (376.5 MHz, $CDCl_3$) δ: –108.15 (m, 1F), –122.58 (m, 2F), –138.78 (m, 1F) ppm. Anal. Calcd for $C_{12}H_7F_3O_2S$: C, 52.94%; H, 2.59%; Found: C, 53.18%; H, 2.47%. HRMS (EI), Calcd for $C_{12}H_7F_3O_2S$ (M^+): m/z = 272.0113; Found: m/z = 272.0157.

1,2,3-Trifluoro-5-(phenylsulfonyl)benzene (4b): 4b was synthesized from **3b** using the same procedures described above for the synthesis of **4a** from **3a.** The product was obtained as a white solid in 95% yield. ^{1}H NMR (400 MHz, $CDCl_3$) δ: 7.93 (m, 2H), 7.66-7.54 (m, 5H) ppm. ^{13}C{^{1}H} NMR(100 MHz, $CDCl_3$) δ: 151.26 (ddd, J_{1CF} = 256.6 Hz, J_{2CF} = 11.0 Hz, J_{3CF} = 3.7 Hz), 143.02 (dt, J_{1CF} = 259.5 Hz, J_{2CF} = 14.7 Hz), 140.10, 137.77 (t, J_{CF} = 3.7 Hz), 134.02, 129.66, 127.82, 112.88 (dd, J_{1CF} = 16.9 Hz, J_{2CF} = 7.3 Hz) ppm. ^{19}F NMR (376.5 MHz, $CDCl_3$) δ: –128.93 (dd, J_1 = 20.0 Hz, J_2 = 4.5 Hz, 2F), –150.69 (tt, J_1 = 20.0 Hz, J_2 = 6.0 Hz, 1F) ppm. Anal. Calcd for $C_{12}H_7F_3O_2S$: C, 52.94%; H, 2.59%; Found: C, 53.09%; H, 2.53%. HRMS (EI), Calcd for $C_{12}H_7F_3O_2S$ (M^+): m/z = 272.0113; Found: m/z = 272.0157.

1,3,5-Trifluoro-2-(phenylsulfonyl)benzene (4c): 4c was synthesized from **3c** using the same procedures described above for the synthesis of **4a** from **3a**. The product was obtained as a white solid in 91% yield. ^{1}H NMR (400 MHz, $CDCl_3$) δ: 8.06 (m, 2 H), 7.65 (m, 1 H), 7.57 (m, 2 H), 6.34 (m, 2 H) ppm. ^{13}C{^{1}H} NMR(100 MHz, $CDCl_3$) δ: 165.80 (dt, J_{1CF} = 258.0 Hz, J_{2CF} = 15.0 Hz), 160.94 (ddd, J_{1CF} = 260.0 Hz, J_{2CF} = 15.0 Hz, J_{3CF} = 6.0 Hz), 141.58, 134.20, 129.34, 127.67, 116.40 (td, J_{1CF} = 15.0 Hz, J_{2CF} = 5.0 Hz), 102.30 (td, J_{1CF} = 26.0 Hz, J_{2CF} = 4.0 Hz) ppm. ^{19}F NMR (376.5 MHz, $CDCl_3$, CF_3COOH) δ: –97.10 (m, 1F), –101.91 (m, 2F) ppm. Anal. Calcd for $C_{12}H_7F_3O_2S$: C, 52.94%; H, 2.59%; Found: C, 52.87%; H, 2.63%. HRMS (EI), Calcd for $C_{12}H_7F_3O_2S$ (M^+): m/z = 272.0113; Found: m/z = 272.0140.

1,2,3,4,5-Pentafluoro-6-(phenylsulfonyl)benzene (4d): **4d** was synthesized from **3d** using the same procedures described above for the synthesis of **4a** from **3a**. The product as a white solid was obtained in 93% yield. ^{1}H NMR (400 MHz, $CDCl_3$) δ: 8.08 (d, J = 8.0 Hz, 2H), 7.72 (t, J = 8.0 Hz, 1H), 7.60 (t, J = 8.0 Hz, 2H) ppm. $^{13}C\{^1H\}$ NMR (100 MHz, $CDCl_3$) δ: 144.83 (dm, J_{CF} = 256.0 Hz), 140.70, 138.13 (dm, J_{CF} = 255.0 Hz), 134.92, 129.67, 127.86, 117.41 (m) ppm (one carbon not observed in ^{1}H decoupled ^{13}C NMR spectrum). $^{13}C\{^{19}F\}$ NMR (100 MHz, $CDCl_3$) δ: 144.72, 144.66, 137.92, 117.39 ppm (carbons with ^{1}H coupling are not listed. From ^{19}F decoupled ^{13}C NMR, carbon at 144.72 ppm is observed). ^{19}F NMR (376.5 MHz, $CDCl_3$) δ: –135.41 (m, 2F), –143.87 (m, 1F), –158.09 (m, 2F) ppm. Anal. Calcd for $C_{12}H_5F_5O_2S$: C, 46.76%; H, 1.64%; Found: C, 46.85%; H, 1.68%. HRMS (EI), Calcd for $C_{12}H_5F_5O_2S$ (M^+): m/z = 307.9924; Found m/z = 307.9870.

3,6-Di-*tert*-butyl-9-(2,5-difluoro-4-(phenylsulfonyl)phenyl)carbazole (DTCz-DPS-1): A mixture of 1,2,4-Trifluoro-5-(phenylsulfonyl)benzene (**4a**) (0.5 g, 1.8 mmol), 3,6-di-*tert*-butylcarbazole (**5a**)[1] (0.5 g, 18 mmol) and K_2CO_3 (5.0 g, 36 mmol) in DMSO (10 mL) was stirred at room temperature for 17 h. Water (70 mL) was added and a white solid was obtained. The solid product was collected by filtration, washed with water, and dried. The crude product was purified by silica gel column chromatography using dichloromethane/hexane (7:3) as eluent. After removal of the solvents under reduced pressure, the product was recrystallized from methanol/water. Solid product was collected by filtration. After vacuum drying, the product was obtained as a white solid (0.82 g, 86%). ^{1}H NMR (400 MHz, $CDCl_3$) δ: 8.11 (m, 5H), 7.71 (m, 1H), 7.62 (tm, J = 8.0 Hz, 2H), 7.45 (dd, J_1 = 8.0 Hz, J_2 = 1.6 Hz, 2H), 7.39 (dd, J_1 = 9.6 Hz, J_2 = 5.6 Hz, 1H), 7.18 (dd, J_1 = 8.8 Hz, J_2 = 2.8 Hz, 2H), 1.45 (s, 18H) ppm. $^{13}C\{^1H\}$ NMR (100 MHz, $CDCl_3$) δ: 155.14 (d, J_{CF} = 252.0 Hz), 152.96 (d, J_{CF} = 253.0 Hz), 144.51, 140.15, 138.01, 134.26, 132.50 (dd, J_{1CF} = 14.0 Hz, J_{2CF} = 8.0 Hz), 129.45, 128.60 (dd, J_{1CF} = 18.0 Hz, J_{2CF} = 6.0 Hz), 128.48, 124.30, 124.14, 118.42 (d, J_{CF} = 25.0 Hz), 117.14 (d, J_{CF} = 23.0 Hz), 116.57, 109.48, 34.82, 31.93 ppm. ^{19}F NMR (376.5 MHz, $CDCl_3$) -110.67 (m, 1F), -118.64 (m, 1F) ppm. Anal. Calcd for $C_{32}H_{31}F_2NO_2S$: C, 72.29%; H, 5.88%;

N, 2.63%; Found: C, 72.08%; H, 5.82%; N, 2.76%. HRMS (EI), Calcd for $C_{32}H_{31}F_2NO_2S$ (M^+): m/z = 531.2044; Found: m/z = 531.2053.

3,6-Di-*tert*-butyl-9-(2,6-difluoro-4-(phenylsulfonyl)phenyl)carbazole (DTCz-DPS-2): **DTCz-DPS-2** was synthesized from **4b** using the same procedures described above for the synthesis of **DTCz-DPS-1** from **4a**. The product was obtained as a white solid in 92% yield. ^{1}H NMR (400 MHz, $CDCl_3$) δ: 8.10 (d, J = 1.6 Hz, 2H), 8.06 (m, 2 H), 7.75 (m, 2H), 7.71 (m, 1H), 7.63 (m, 2H), 7.45 (dd, J_1 = 8.4 Hz, J_2 = 1.6 Hz, 2H), 6.99 (dt, br, J_1 = 8.4 Hz, J_2 = 1.6 Hz, 2H), 1.45 (s, 18H) ppm. ^{13}C{^{1}H} NMR(100 MHz, $CDCl_3$) δ: 158.80 (dd, J_{1CF} = 260.2 Hz, J_{2CF} = 4.4 Hz), 144.09, 142.28 (t, J_{CF} = 7.3 Hz), 139.97, 137.99, 134.17, 129.76, 128.12, 124.03, 123.93, 119.68 (t, J_{CF} = 16.2 Hz), 116.48, 112.41 (d, J_{CF} = 27.1 Hz), 109.35, 34.73, 31.90 ppm. ^{19}F NMR (376.5 MHz, $CDCl_3$) δ: –109.08 (d, J = 6.8 Hz, 2F) ppm. Anal. Calcd for $C_{32}H_{31}F_2NO_2S$: C, 72.29%; H, 5.88%; N, 2.63%; Found: C, 72.12%; H, 5.85%; N, 2.69%, HRMS (EI), calcd for $C_{32}H_{31}F_2NO_2S$ (M^+): m/z = 531.2044; Found: m/z = 531.2052.

3,6-Di-*tert*-butyl-9-(2,3,5,6-tetrafluoro-4-(phenylsulfonyl)phenyl)carbazole (DTCz-DPS-3): DTCz-DPS-3 was synthesized from **4d** using the same procedures described above for the synthesis of **DTCz-DPS-1** from **4a**. The product as a light yellow solid in 75% yield was obtained. ^{1}H NMR (400 MHz, $CDCl_3$) δ: 8.20 (d, J = 8.0 Hz, 2H), 8.10 (d, J = 1.6 Hz, 2H), 7.76 (m, 1H), 7.67 (m, 2H), 7.47 (dd, J_1 = 8.4 Hz, J_2 = 1.6 Hz, 2H), 7.02 (dt, br, J_1 = 8.4 Hz, J_2 = 1.6 Hz, 2H), 1.45 (s, 18H) ppm. ^{13}C{^{1}H} NMR (100 MHz, $CDCl_3$) δ: 144.92 (dd, J_{1CF} = 255.0 Hz, J_{2CF} = 13.0 Hz), 144.93, 143.74 (dd, J_{1CF} = 262.0 Hz, J_{2CF} = 13.0 Hz), 140.78, 137.51, 134.97, 129.74, 128.18, 124.49, 124.26, 122.39 (t, J = 13.0 Hz), 120.03 (t, J = 13.0 Hz), 116.68, 109.63, 34.84, 31.91 ppm. ^{19}F NMR (376.5 MHz, $CDCl_3$) δ: –135.40 (m, 2F), –138.89 (m, 2F) ppm. Anal. Calcd for $C_{32}H_{29}F_4NO_2S$: C, 67.71%; H, 5.15%; N, 2.47%; Found: C, 67.62%; H, 5.09%; N, 2.54%. HRMS (EI) Calcd for $C_{32}H_{29}F_4NO_2S$ (M^+): m/z = 567.1855, Found: m/z = 567.1856.

3,6-Di-*tert*-butyl-9-(3,5-difluoro-4-(phenylsulfonyl)phenyl)carbazole (DTCz-DPS-0): DTCz-DPS-0 was synthesized from **4c** using the same procedures described above for the synthesis of **DTCz-DPS-1** from **4a**. The product was obtained as a white solid in 32% yield. ^{1}H NMR (400 MHz, $CDCl_3$) δ: 8.17 (d, J = 8.0 Hz, 2H), 8.09 (t, J = 1.6 Hz, 2H), 7.70 (m, 1H), 7.62 (m, 2H), 7.47 (m, 4H), 7.28 (d, J = 10.0 Hz, 2H), 1.45 (s, 18 H) ppm. ^{13}C{^{1}H} NMR (125 MHz, $CDCl_3$) δ: 160.87 (dd, J_{1CF} = 258.6 Hz, J_{2CF} = 6.3 Hz), 145.13 (t, J_{CF} = 12.5 Hz), 145.03, 141.88, 137.44, 134.16, 129.40, 127.88, 124.61, 124.35, 116.71, 116.24 (t, J_{CF} = 16.3 Hz), 109.83 (dd, J_{1CF} = 26.3 Hz, J_{2CF} = 3.8 Hz), 109.38, 34.84, 31.89 ppm. ^{19}F NMR (376.5 MHz, $CDCl_3$) δ: -103.90 (d, J = 9.4 Hz, 2F) ppm. Anal. Calcd for $C_{32}H_{31}F_2NO_2S$: C, 72.29%; H, 5.88%; N, 2.63%; Found: C, 72.20%; H, 5.80%; N, 2.72%. HRMS (EI), Calcd for $C_{32}H_{31}F_2NO_2S$ (M^+): m/z = 531.2044; Found: m/z = 531.2045.

9-(2,6-Difluoro-4-(phenylsulfonyl)phenyl)carbazole (Cz-DPS-1): Cz-DPS-1 was synthesized using the same procedures described above for the synthesis of **DTCz-DPS-1** using carbazole in place of 3,6-di-*tert*-butylcarbazole and **4b** in place of **4a**. The product was obtained as a white solid in 95% yield. ^{1}H NMR (400 MHz, $CDCl_3$) δ: 8.11 (m, 2H), 8.07 (m, 2H), 7.75 (m, 2H), 7.71 (m, 1 H), 7.64 (m, 2H), 7.42 (m, 2H), 7.33 (m, 2H), 7.08 (m, br, 2H) ppm. ^{13}C{^{1}H} NMR(100 MHz, $CDCl_3$) δ: 158.71 (dd, J_{1CF} = 260.3 Hz, J_{2CF} = 4.4 Hz), 142.98 (t, J_{CF} = 7.3 Hz), 139.92, 139.64, 134.28, 129.84, 128.19, 126.40, 124.09, 121.19, 120.52, 119.26 (t, J_{CF} = 16.1 Hz), 112.56 (dd, J_{1CF} = 19.8 Hz, J_{2CF} = 6.6 Hz), 109.92 ppm. ^{19}F NMR (376.5 MHz, $CDCl_3$) δ: -109.02 (d, J = 5.6 Hz, 2F) ppm. Anal. Calcd for $C_{21}H_{15}F_2NO_2S$: C, 68.72%; H, 3.60%; N, 3.34%; Found: C, 68.81%; H, 3.57%; N, 3.38%. HRMS (EI), Calcd for $C_{21}H_{15}F_2NO_2S$ (M^+): m/z = 419.0792; Found m/z = 419.0791.

9-(2,3,5,6-Tetrafluoro-4-(phenylsulfonyl)phenyl)-9H-carbazole (Cz-DPS-2): Cz-DPS-2 was synthesized using the same procedures described above for the synthesis of **DTCz-DPS-1** using carbazole in place of 3,6-di-*tert*-butylcarbazole and **4d** in place of **4a.**. The product as a pale-yellow solid in 86% was obtained. ^{1}H NMR (400 MHz, $CDCl_3$) δ: 8.20 (d, *J* = 8.0 Hz, 2H), 8.11 (dt, J_1 = 8.0 Hz, J_2 = 0.8 Hz, 2H), 7.77 (m, 1H), 7.68 (tt, J_1 = 8.0 Hz, J_2 = 0.8 Hz, 2H), 7.45 (td, J_1 = 8.0 Hz, J_2 = 1.2 Hz, 2H), 7.36 (td, J_1 = 8.0 Hz, J_2 = 0.8 Hz, 2H), 7.11 (dt, br, *J* = 8.0 Hz, 2H) ppm. ^{13}C{^{1}H} NMR (125 MHz, $CDCl_3$) δ: 144.94 (dd, J_{1CF} = 260.0 Hz, J_{2CF} = 13.9 Hz), 143.94 (dd, J_{1CF} = 256.3 Hz, J_{2CF} = 13.8 Hz), 140.66, 139.13, 135.04, 129.77, 128.20, 126.69, 124.46, 121.86, 120.80, 110.09 ppm (Two carbons not observed in the ^{1}H decoupled ^{13}C NMR). ^{13}C{^{19}F} NMR (100 MHz, $CDCl_3$) δ: 144.78, 143.80, 121.85, 120.62 ppm (Carbons with ^{1}H coupling are not listed. From ^{19}F decoupled ^{13}C NMR, the two carbons missing in the ^{1}H-decoupled ^{13}C spectrum are seen at 121.76 ppm and 120.46 ppm). ^{19}F NMR (376.5 MHz, $CDCl_3$) δ: –135.09 (m, 2F), –138.68 (m, 2F) ppm. Anal. Calcd for $C_{24}H_{13}F_4NO_2S$: C, 63.30%; H, 2.88%; N, 3.08%; Found: C, 63.36%; H, 2.79%; N, 3.17%. HRMS (EI) Calcd for $C_{24}H_{13}F_4NO_2S$ (M^+): m/z = 455.0603; Found: m/z = 455.0606.

9'-(2,6-Difluoro-4-(phenylsulfonyl)phenyl)-9'*H*-9,3':6',9''-tercarbazole (3Cz-DPS-1): 3Cz-DPS-1 was synthesized using the same procedures described above for the synthesis of **DTCz-DPS-1** using 3,6-di(carbazol-9-yl)carbazole (**5c**)[2] and **4b** in place of carbazole and **4a**, repsectively. The product was obtained as a white solid in 95% yield. ^{1}H NMR (400 MHz, $CDCl_3$) δ: 8.27 (d, J = 2 Hz, 2H), 8.15 (m, 4H), 8.11 (m, 2H), 7.89 (m, 2H), 7.75 (m, 1H), 7.66 (m, 4H), 7.35 (m, br, 10H), 7.26 (m, 4H) ppm. ^{13}C{^{1}H} NMR (125 MHz, $CDCl_3$) δ: 159.05 (dd, J_{1CF} = 260.0 Hz, J_{2CF} = 3.8 Hz), 143.97 (t, J_{CF} = 7.5 Hz), 141.62, 139.82, 139.40, 134.46, 131.68, 129.94, 128.31, 126.70, 126.00, 124.90, 123.26, 120.37, 119.95, 119.90, 118.66 (t, J_{CF} = 16.3 Hz), 112.81 (d, J_{1CF} = 26.3 Hz), 111.50, 109.67 ppm. ^{19}F NMR (376.5 MHz, $CDCl_3$) δ: –108.98 (d, *J* = 7.2 Hz, 2F) ppm. Anal. Calcd for $C_{48}H_{29}F_2N_3O_2S$: C, 76.89%; H, 3.90%; N, 5.60%; Found: C, 77.12%; H, 3.75%; N, 5.66%. HRMS (EI), Calcd for $C_{48}H_{29}F_2N_3O_2S$ (M^+): m/z = 749.1949; Found m/z = 749.1965.

9'-(2,3,5,6-Tetrafluoro-4-(phenylsulfonyl)phenyl)-9'*H*-9,3':6',9''-tercarbazole (3Cz-DPS-2): 3Cz-DPS-2 was synthesized using the same procedures described above for the synthesis of **DTCz-DPS-1** using 3,6-di(carbazol-9-yl)carbazole (**5c**) and **4d** in place of carbazole and **4a**, respectively. The product was obtained as a light-yellow solid in 76% yield. ^{1}H NMR (400 MHz, $CDCl_3$) δ: 8.27 (d, J = 2.0 Hz, 2H), 8.24 (d, J = 8.0 Hz, 2H), 8.15 (d, J = 8.0 Hz, 4H), 7.80 (m, 1H), 7.71 (d, J = 8.0 Hz, 2H), 7.67 (dd, J_1 = 8.0 Hz, J_2 = 2.0 Hz, 2H), 7.37 (m, 10H), 7.27 (m, 4 H) ppm. ^{13}C{^{1}H} NMR (125 MHz, $CDCl_3$) δ: 144.99 (dd, J_{1CF} = 273.8 Hz, J_{2CF} = 11.3 Hz), 144.01 (dd, J_{1CF} = 255.0 Hz, J_{2CF} = 11.3 Hz), 141.51, 140.58, 138.82, 135.20, 132.29, 129.85, 128.31, 126.91, 126.03, 125.29, 123.31, 121.63 (t, J = 11.3 Hz), 121.22 (t, J = 11.3 Hz), 120.40, 119.98, 111.64, 109.59 ppm. ^{19}F NMR (376.5 MHz, $CDCl_3$) δ: –134.22 (m, 2F), –138.55 (m, 2F) ppm. Anal. Calcd for $C_{48}H_{27}F_4N_3O_2S$: C, 73.37%; H, 3.46%; N, 5.35%; Found: C, 73.42%; H, 3.31%; N, 5.39%. HRMS (MALDI), Calcd for $C_{48}H_{27}F_4N_3O_2S$ (M^+), m/z = 785.1760; Found: m/z = 785.1786.

9-(4-(Phenylthio)phenyl)carbazole (8): A mixture of 9-(4-bromophenyl)carbazole (**6**) (13.8 g, 43 mmol), benzenethiol (**7**) (4.7 g, 42 mmol) and potassium carbonate (20.0 g, 144 mmol) in *N,N*-dimethylformamide DMF (100 mL) was stirred at 150 °C for 24 h. After cooling to room temperature, water (200 mL) was added. The white solid product was obtained and collected by filtration. The crude product was purified by silica gel column chromatography using dichloromethane/hexanes (1:9) as eluent. After removal of solvents, the white solid product was obtained (14.2 g, 96%). ^{1}H NMR (400 MHz, CDCl3,) δ: 8.13 (dt, J_1 = 7.6 Hz, J_2 = 1.2 Hz, 2H), 7.52-7.46 (m, 6H), 7.42-7.38 (m, 6H), 7.36-7.27 (m, 3H) ppm. ^{13}C{^{1}H} NMR (100 MHz, $CDCl_3$) δ: 140.68, 136.24, 135.73, 134.56, 132.10, 131.33, 129.46, 127.78, 127.62, 125.95, 123.41, 120.31, 120.05, 109.69 ppm. Anal. Calcd for $C_{24}H_{17}NS$: C, 82.02; H, 4.88; N, 3.99. Found: C, 81.72; H, 4.81; N, 4.00. HRMS (EI), Calcd for $C_{24}H_{17}NS$ (M^+): m/z = 551.1082; Found: m/z = 551.1086.

9-(4-(Phenylsulfonyl)phenyl)carbazole (Cz-DPS-0): A mixture of 9-(4-(phenylthio)phenyl)-carbazole (**8**) (1.0 g, 2.9 mmol) and *m*-chloroperbenzoic acid (1.1 g, 6 mmol) in dichloromethane (50 mL) was stirred at room temperature for 20 h. After removal of dichloromethane, the crude product was purified by silica gel column chromatography using dichloromethane as eluent. After removal of solvents and drying, the white solid product was obtained (0.97 g, 89%). ^{1}H NMR (400 MHz, CDCl3,) δ: 8.16 (d, J = 8.8 Hz, 2H), 8.11 (dt, J_1 = 7.6 Hz, J_2 = 1.2 Hz, 2H), 8.06-8.03 (m, 2H), 7.72 (d, J = 8.8 Hz, 2H), 7.64-7.56 (m, 3H), 7.43-7.37 (m, 4H), 7.30 (td, J_1 = 7.6 Hz, J_2 = 1.2 Hz, 2H) ppm. ^{13}C{^{1}H} NMR (100 MHz, $CDCl_3$) δ: 142.44, 141.35, 139.94, 139.71, 133.49, 129.56, 129.50, 127.85, 127.08, 126.32, 123.94, 120.92, 120.53, 109.60 ppm. Anal. Calcd for $C_{24}H_{17}NO_2S$: C, 75.17; H, 4.47; N, 3.65. Found: C, 75.13; H, 4.44; N, 3.62. HRMS (EI), Calcd for $C_{24}H_{17}NO_2S$ (M^+): m/z = 383.0980. Found: m/z = 383.0977.

1-(3,6-Di(carbazol-9-yl)carbazol-9yl)-4-(phenylsulfonyl)benzene (3Cz-DPS-0): A reaction mixture of 1-chloro-4-(phenylsulfonyl)benzene (0.60 g, 2.4 mmol), 3,6-di(carbazol-9-yl)carbazole (**5c**) (1.0 g, 2.0 mmol) and potassium carbonate (4.0 g, 29 mmol) in DMSO (8.0 mL) was stirred at 170 °C under nitrogen for 24 h. After cooling down to room temperature, water (75 mL) was added into the reaction mixture, and the resulting yellow solid was obtained and collected with filtration. The solid was washed with water and methanol. After dry, the product was purified with silica gel chromatography column using dichloromethane/hexanes (7:3) as eluent. After removal of solvents, white solid product was obtained. The solid was re-dissolved in dichloromethane and then methanol (50 ml) was added, dichloromethane was removed under reduced pressure and the product was obtained as a white solid by filtration. After vacuum drying, the product was obtained as a white solid in (1.03 g, 71%). ^{1}H NMR (400 MHz, $CDCl_3$, δ): 8.29 (d, J = 8.4 Hz, 2 H), 8.26 (d, J = 2.0 Hz, 2 H), 8.15 (d, J = 7.6 Hz, 4 H), 8.09 (d, J = 8.4 Hz, 2 H), 7.91 (d, J = 8.4 Hz, 2 H), 7.69-7.59 (m, 7 H), 7.41-7.36 (m, 8 H), 7.28 (td, J_1 = 7.6 Hz, J_2 = 2.0 Hz, 4 H) ppm. ^{13}C NMR (100 MHz, $CDCl_3$, δ): 141.76, 141.56, 141.12, 140.75, 139.72, 133.63, 131.24, 129.89, 129.56, 127.92, 127.32, 126.54, 125.93, 124.57, 123.19, 120.34, 119.86, 119.82, 111.14, 109.54 ppm. Anal. Calcd for $C_{48}H_{31}N_3O_2S$: C, 80.76; H, 4.38; N, 5.89. Found: C, 80.79; H, 4.27; N, 5.85. HRMS (MALDI), Calcd for $C_{48}H_{31}N_3O_2S$ (M^+): *m/z* = 713.2137; Found: m/z = 713.2225.

Density functional theory calculations

Supplementary Table 1. Excited state energies and oscillator strength of the fluorescent $S_1 \rightarrow S_0$ transitions (*f*) predicted by TD-DFT LC-BLYP/6-31G(d).

Compound	S_1 (eV)	S_2 (eV)	T_1 (eV)	*f*
DTCz-DPS-1	3.98	4.92	2.96	0.341
DTCz-DPS-2	3.87	4.93	2.97	0.310
DTCz-DPS-3	3.41	4.93	2.96	0.199

Supplementary Table 2. Contribution to zero-field splitting *D* parameter in each triplet configuration from the spin-spin and the spin-orbit interactions, as predicted by DFT PBE0/6-31G(d).

	^{3}LE *D* parameter contribution (mT)		^{3}CT *D* parameter contribution (mT)	
Compound	Spin-spin	Spin-orbit	Spin-spin	Spin-orbit
DTCz-DPS-1	−43	−71	/	/
DTCz-DPS-2	−40	−70	−0.6	−75
DTCz-DPS-3	−36	−75	−20	−75

Supplementary Table 3. Calculations of $\langle \Psi_{^1CT} | \hat{H}_{SO} | \Psi_{^3LE} \rangle$ in the *x*, *y*, and *z* planes at the optimised ^{3}LE geometries by TDDFT LC-BLYP/6-31G(d) with the zeroth-order regular approximation.

	$\langle \Psi_{^1CT} \| \hat{H}_{SO} \| \Psi_{^3LE} \rangle$ cm^{-1}		
Compound	*x*	*y*	*z*
DTCz-DPS-1	0.31	0.03	0.01
DTCz-DPS-2	0.27	0.00	0.02
DTCz-DPS-3	0.32	0.01	0.03

Electron spin resonance

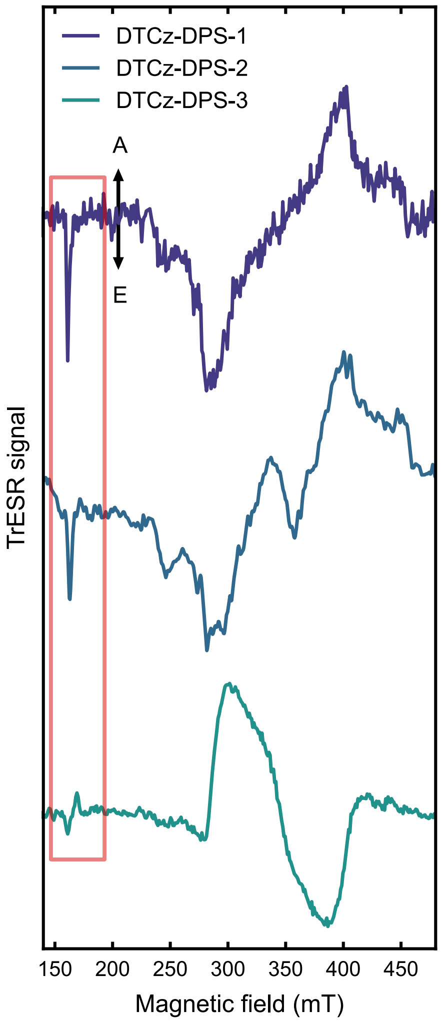

Supplementary Figure 4. **Electron spin resonance half-field** Spin-polarised trESR signal of DTCz-DPS-1 (top), DTCz-DPS-2 (middle), and DTCz-DPS-3 (bottom) collected at 30 K in deoxygenated toluene (500 µM), recorded 2 µs after 355 nm laser excitation and integrated over 1 µs.

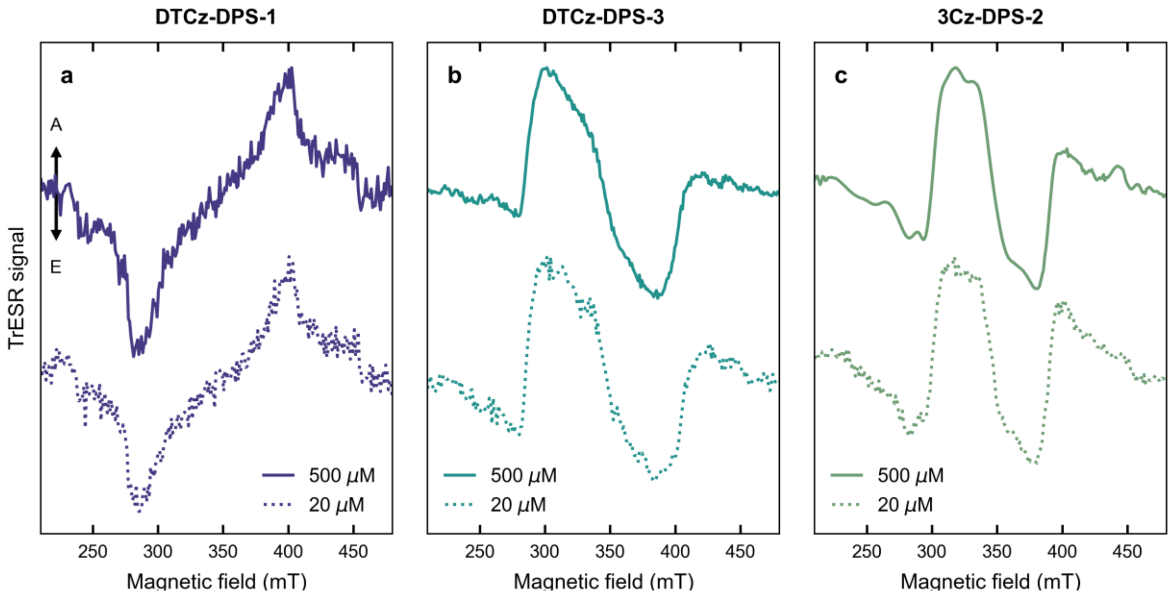

Supplementary Figure 5. **Electron spin resonance of frozen diluted solutions** Spin-polarised trESR signal of (a) DTCz-DPS-1, (b) DTCz-DPS-3 and (c) 3Cz-DPS-2 collected at 30 K in deoxygenated toluene (500 µM top, 20 µM bottom), recorded 2 µs after 355 nm laser excitation and integrated over 1 µs.

Supplementary Table 4. Electron spin resonance fit parameters: ZFS parameters (*D* and *E*) and spin sublevel populations ($P_{x,y,z}$) of ^{3}LE and ^{3}CT obtained by simulation of the trESR signal detected at 30 K in degassed toluene. The $P_{x,y,z}$ uncertainty is ± 0.1 in all cases except for Cz-DPS-1 ^{3}LE, which has an uncertainty ± 0.05. The weighting factor corresponds to the contribution of each ^{3}LE or ^{3}CT simulation to its total simulated spectrum.

	^{3}LE PARAMETERS			^{3}CT PARAMETERS		
**Compound**	**Experiment D, E (mT)**	$P_{x,y,z}$	**Weight**	**Experiment D, E (mT)**	$P_{x,y,z}$	**Weight**
Cz-DPS-1	−114, −9	0.40, 0.00, 0.60	1.0	/	/	/
Cz-DPS-2	−114, −9	0.4, 0.0, 0.6	0.65	−72, −13	0.4, 0.6, 0.0	0.35
3Cz-DPS-1	−105, −10	0.4, 0.0, 0.6	1.0	/	/	/
3Cz-DPS-2	−112, −6	0.4, 0.0, 0.6	0.33	−45, −10	0.5, 0.5, 0.0	0.67
3Cz-DPS-2 in MeTHF	−112, −6	0.4, 0.0, 0.6	0.70	−45, −10	0.5, 0.5, 0.0	0.30

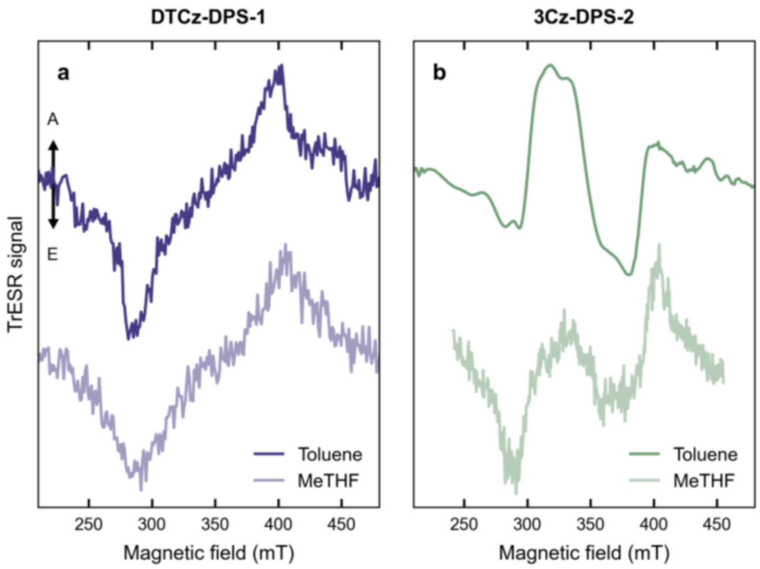

Supplementary Figure 6. **Electron spin resonance in frozen toluene and 2-methyltetrahydrofuran solutions** Spin-polarised trESR signal of (a) DTCz-DPS-1 and (b) 3Cz-DPS-2 collected at 30 K in deoxygenated toluene 500 µM (top) and 2-methyltetrahydrofuran (MeTHF) 500 µM (bottom), recorded 2 µs after 355 nm laser excitation and integrated over 1 µs.

We consider that the difference between toluene and MeTHF trESR spectra can be understood using the following rationale. We expect that changing the molecular environment (solvent) shifts CT energies. When changing from toluene to MeTHF the ^{3}CT intensity decreases in (b) 3Cz-DPS-2; the 3CT weighting decreases from 0.67 in toluene to 0.30 in MeTHF (Supplementary Table 4). This could suggest that solvent-dependent changes in charge-transfer energies increase the ^{3}LE–^{3}CT gap, reducing the strength of the ^{3}LE–^{3}CT vibronic coupling and hence reducing the transfer from ^{3}LE to ^{3}CT that we observe in 3Cz-DPS-2. In toluene, the larger ^{3}LE–^{3}CT gap molecule DTCz-DPS-1 only exhibits a ^{3}LE signal. In MeTHF the DTCz-DPS-1 trESR spectrum remains purely 3LE because a solvent-induced increase in the ^{3}LE–^{3}CT gap only acts to decrease any ^{3}LE–^{3}CT vibronic coupling.

Additional compounds: trESR, photophysics and DFT

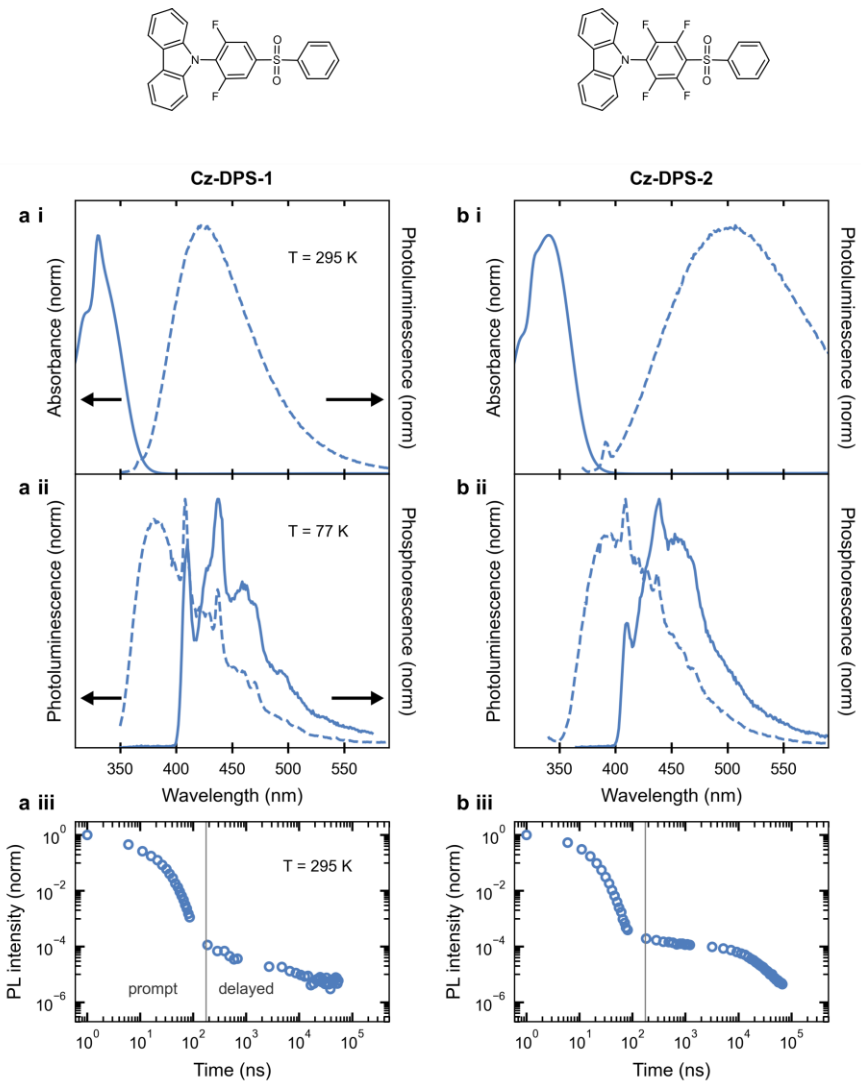

Supplementary Figure 7. **Additional compound structures (top) and photophysical spectra**. (a) Cz-DPS-1 and (b) Cz-DPS-2. (i) Normalized absorbance (solid line) and photoluminescence (dashed line) spectra in deoxygenated toluene at 295 K. (ii) Normalized steady-state photoluminescence (dashed line) and gated (0.5 ms) phosphorescence (solid line) spectra in deoxygenated toluene at 77 K. (iii) Normalised integrated photoluminescence kinetics in deoxygenated toluene at 295 K.

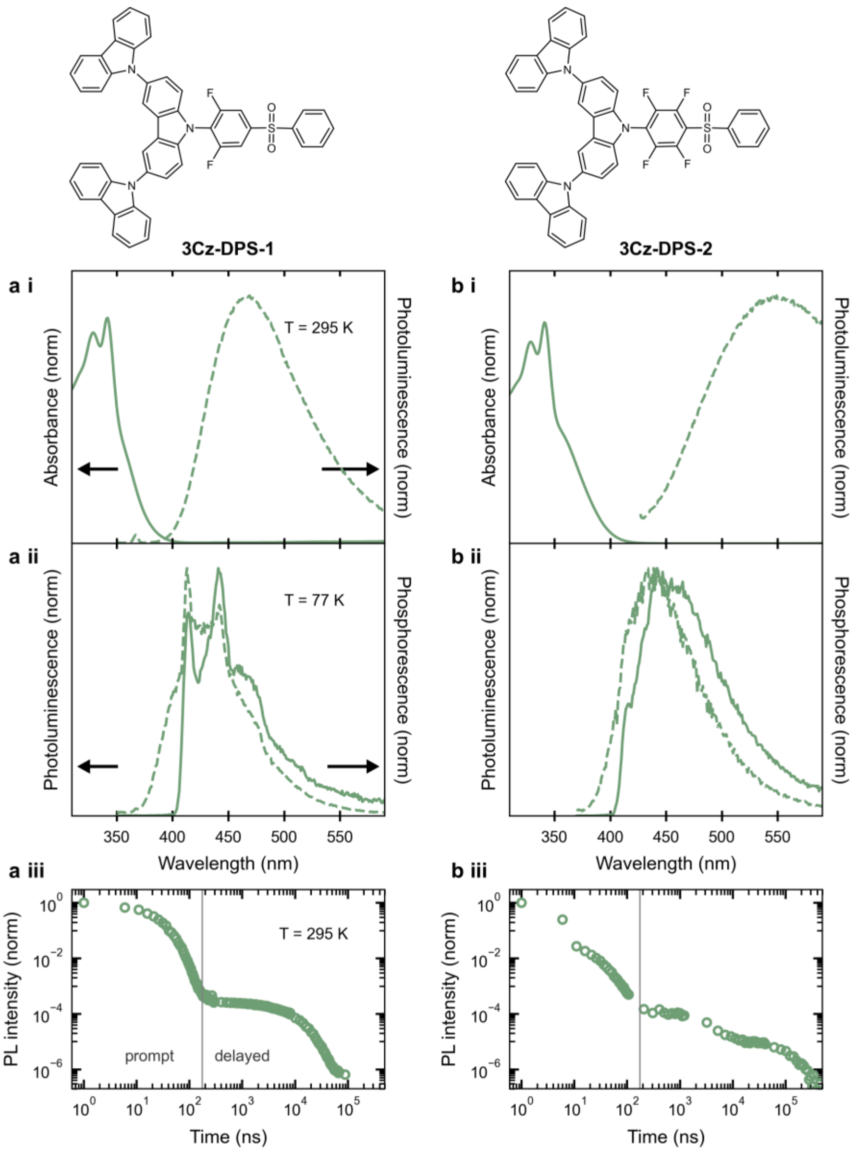

Supplementary Figure 8. **Additional compound structures (top) and photophysical spectra**. (a) 3Cz-DPS-1 and (b) 3Cz-DPS-2. (i) Normalized absorbance (solid line) and photoluminescence (dashed line) spectra in deoxygenated toluene at 295 K. (ii) Normalized steady-state photoluminescence (dashed line) and gated (0.5 ms) phosphorescence (solid line) spectra in deoxygenated toluene at 77 K. (iii) Normalised integrated photoluminescence kinetics in deoxygenated toluene at 295 K.

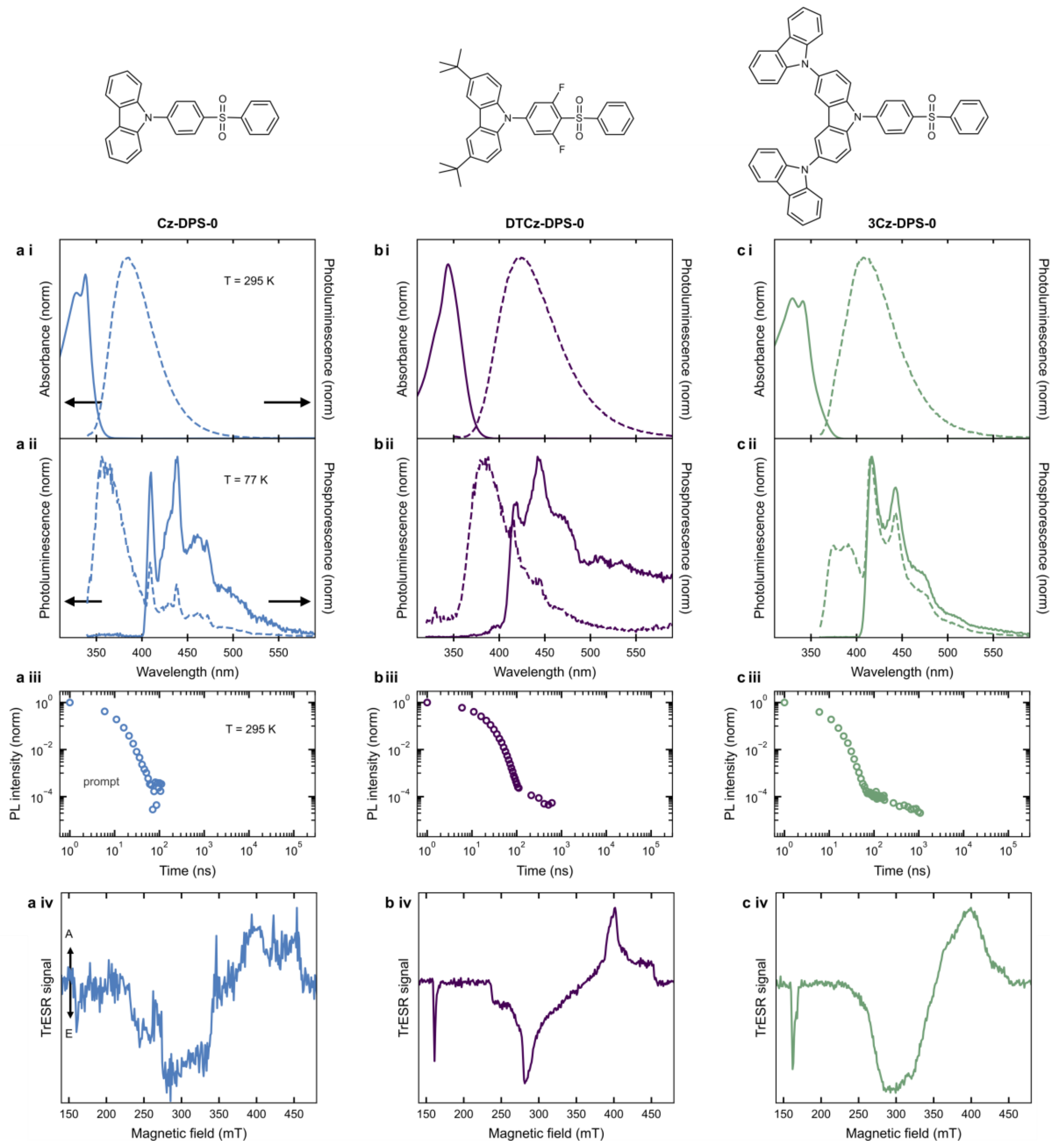

Supplementary Figure 9. **Additional compound structures (top) and photophysical and trESR spectra**. (a) Cz-DPS-0, (b) DTCz-DPS-0, and (d) 3Cz-DPS-0. (i) Normalized absorbance (solid line) and photoluminescence (dashed line) spectra in deoxygenated toluene at 295 K. (ii) Normalized steady-state photoluminescence (dashed line) and gated (0.5 ms) phosphorescence (solid line) spectra in deoxygenated toluene at 77 K. (iii) Normalised integrated photoluminescence kinetics in deoxygenated toluene at 295 K. Delayed fluorescence was recorded and plotted until it could no longer be distinguished from the noise level. (iv) Spin-polarised trESR signal collected at 30 K in deoxygenated toluene (500 µM), 2 µs after 355 nm laser excitation and integrated over 1 µs.

Supplementary Table 5. Experimental excited state and kinetic properties.

Compound	S_1 (eV)[a]	T_1 (eV)[a]	λ_{PL} (nm)[b]	% delayed[d]
Cz-DPS-1	3.55	3.09	423	4
Cz-DPS-2	3.49	3.09	500	11
3Cz-DPS-1	3.29	3.06	466	10
3Cz-DPS-2	3.18	3.05	550	26

[a]Energetic onset of photoluminescence (S1) and phosphorescence (T1) in deoxygenated toluene at 77 K. [b]Photoluminescence charge-transfer transition peak maxima measured in deoxygenated toluene at 295 K. [c]Contribution of delayed fluorescence component to total photoluminescence from integrated time-resolved photoluminescence measured in deoxygenated toluene at 295 K.

Supplementary Table 6. Excited state energies and oscillator strength of the fluorescent $S_1 \rightarrow S_0$ transitions (*f*) predicted by TD-DFT LC-BLYP/6-31G(d). Calculations of $\langle \Psi_{^1CT} | \hat{H}_{SO} | \Psi_{^3LE} \rangle$ in the *x*, *y*, and *z* planes at the optimised ^{3}LE geometries by TDDFT LC-BLYP/6-31G(d) with the zeroth-order regular approximation.

Compound	S_1 (eV)	T_1 (eV)	*f*	$\langle \Psi_{^1CT} \| \hat{H}_{SO} \| \Psi_{^3LE} \rangle$ cm^{-1}		
				x	*y*	*z*
Cz-DPS-1	4.01	2.96	0.320	0.29	0.00	0.02
Cz-DPS-2	3.54	2.96	0.212	0.38	0.01	0.03
3Cz-DPS-1	4.11	2.96	0.585	0.36	0.00	0.01
3Cz-DPS-2	3.56	2.95	0.342	0.38	0.01	0.02

Photophysics

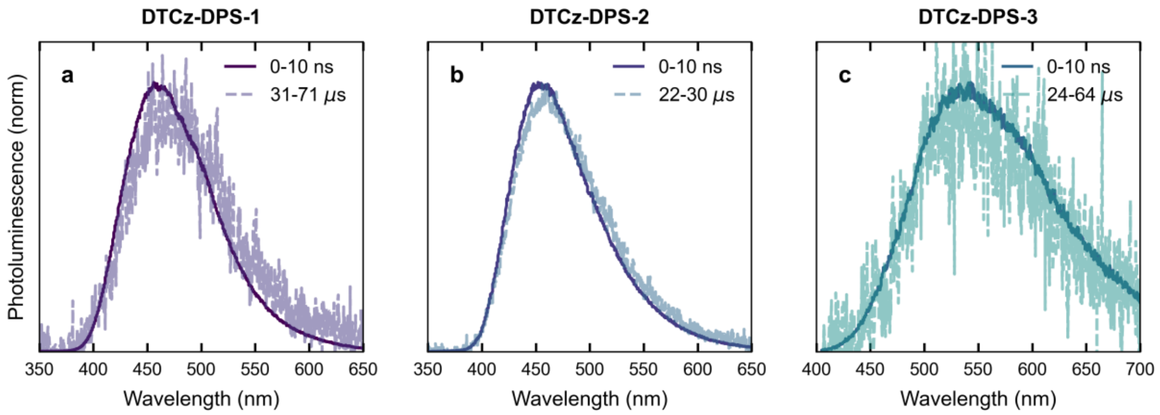

Supplementary Figure 10. **Early and late time photoluminescence** Time-resolved photoluminescence spectra of prompt (solid line) versus delayed (dashed line) fluorescence. (a) DTCz-DPS-1, (b) DTCz-DPS-2 and (c) DTCz-DPS-3 in deoxygenated toluene at 295 K.

Electroluminescence performance

To demonstrate the practical potential of this class of emitters we fabricated OLEDs by solution-processing methods, employing either DTCz-DPS-1 or DTCz-DPS-3 as the dopant in the emissive layer. The simple device structure employed was: ITO / PEDOT:PSS (40 nm) / DTCz-DPS-1 or -3 in o-CBP (30 nm) / TSPO1 (50 nm) / LiF (0.8 nm) / Al (100 nm) (Supplementary Fig 11a). Electroluminescence, external quantum efficiency, current density-voltage and radiance-voltage characteristics are shown in Supplementary Fig 11. Device structure optimisation was not pursued.

The device performances exhibited by each emitter are not compared because this study is concerned solely with revealing the molecular emission mechanism; discussion of device performance would involve considerations of a range of device properties that are unrelated to the fundamental emission mechanism. For example, one reason why drawing correlation between molecular structure and OLED performance is problematic is that the optical energy relative to the host material differs for each emitter. On the other hand, device photophysical properties are fundamental to OLED performance, which is why understanding the nature of the underlying emission mechanisms is essential for OLED development.

Supplementary Table 7. OLED device performance

Compound	EL peak (nm)	EQE peak (%)	Turn-on (V)
DTCz-DPS-1	440	4.3	3.6
DTCz-DPS-3	508	6.0	3.4

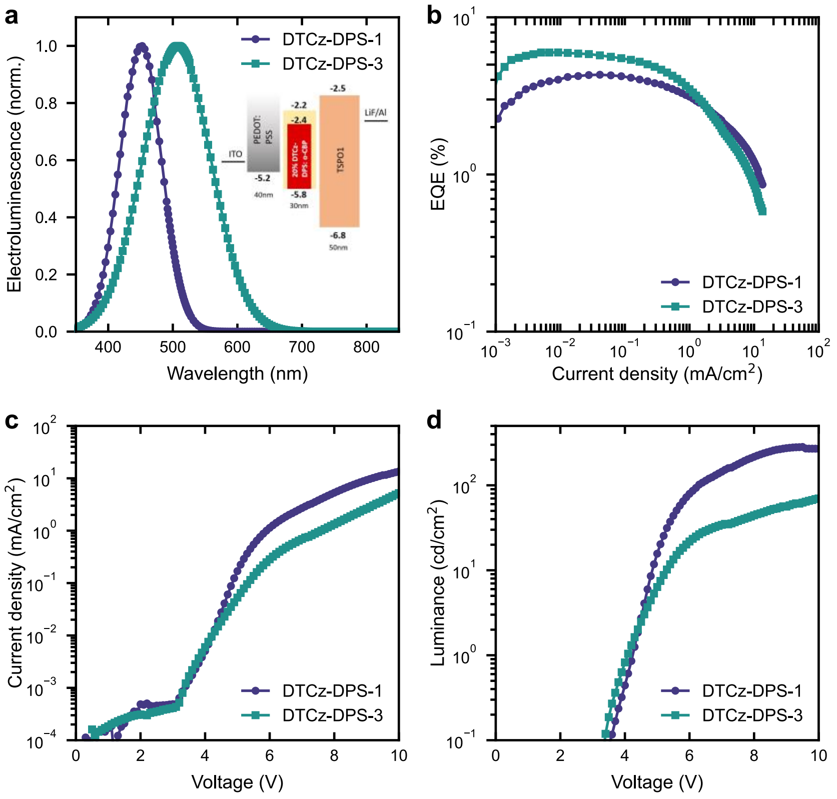

Supplementary Figure 11. **OLED device structure and performance. a** Electroluminescence spectra (inset: device structure), **b** external quantum efficiency versus current density, **c** current density-voltage characteristic, **d** radiance-voltage characteristic of devices with DTCz-DPS-1 or -3 as the EML dopant.

Solution-processed device fabrication: Devices were carried out by solution processing with a forward configuration as follows: ITO/PEDOT:PSS/EML/ETL/LiF/Al. Indium tin-oxide (ITO; WF ~ 4.8 eV) coated glass substrates were used and subsequently cleaned by sonication in acetone and 2-propanol for 10 minutes respectively, followed by O_2 plasma treatment for 10 minutes. PEDOT: PSS (Clevios CH4083, LumTech Taiwan) was spin-casted on top of the ITO under ambient conditions and annealed on a hot plate at 160 °C for 20 minutes, forming a 40 nm-thick film. The PEDOT: PSS-coated substrates were then transferred to a nitrogen-filled glovebox to conduct the following solution processes. The 40 nm-thick emitting layer (EML) of o-CBP (LumTech Taiwan) doped with 20 wt.% of DTCz-DPS-1 or -3 in toluene was spin-coated on top of the PEDOT:PSS layer and baked on a hot plate at 90 °C for 10 min to remove any solvent present. The samples were then transferred to a vacuum deposition system. 50 nm-thick of TSPO1 (Ossila) were deposited as electron transport layers. Finally, 0.8 nm-thick LiF (99.99%, Sigma-Aldrich) and 100 nm-thick aluminium were subsequently deposited by thermal evaporation under high vacuum (< 3×10^{-7} mbar).

Characterisation of OLED device performance: The forward-viewing current–voltage–luminance characteristics of these OLED devices were measured using a Keithley 2400 source meter, Keithley 2000 multimeter and a calibrated Si photodiode (from RS components), which was placed at a distance of 2 cm from the devices. External quantum efficiencies (EQE) were calculated from on-axis irradiance assuming a Lambertian emission profile and accounting for photodiode quantum efficiency across the electroluminescence spectrum. The electroluminescence spectra were obtained by a fibre spectrometer (Flame-S-VIS-NIR-ES, Ocean Optics). All the measurements were carried out at room temperature under ambient conditions.
